# Spontaneous Insertion of Aβ42 Dimers but Not Monomers into a Cholesterol-Rich Lipid Bilayer

**DOI:** 10.1021/acschemneuro.5c00942

**Published:** 2026-05-22

**Authors:** Rachit Pandey, Thomas Ruggiero, Brian Andrews, Brigita Urbanc

**Affiliations:** Physics Department, 6527 Drexel University, 3141 Chestnut St, Philadelphia, Pennsylvania 19104, United States

**Keywords:** Alzheimer’s disease, Amyloid β-protein (Aβ), Lipid bilayer, Cholesterol, Molecular dynamics

## Abstract

The leading Alzheimer’s disease (AD) hypothesis posits that oligomers formed by amyloid β-protein (Aβ), in particular 42-residue-long Aβ42, interact with a cellular membrane, causing a cascade of events leading to neurodegeneration. The modes of Aβ42-lipid interactions are not well understood. Here, we use explicit-solvent all-atom molecular dynamics (MD) to demonstrate that Aβ42 monomers interact with lipids differently than Aβ42 dimers. In our simulations, lipids in the absence and presence of Aβ42 form a lipid bilayer with a cholesterol-rich domain, resembling a lipid raft. Whereas lipids stabilize the Aβ42 monomer structure, they partially destabilize Aβ42 dimers. Unlike monomers, which interact exclusively with solvent-exposed lipid tails on one side of a bilayer, dimers exhibit additional modes of interactions with lipids, including spontaneous insertion into the cholesterol-rich domain of a bilayer and carpeting, thereby disrupting the lipid bilayer structure. Our findings provide a mechanistic explanation for why Aβ42 monomers are nontoxic and reveal that Aβ42 oligomer-induced toxicity emerges already at the stage of Aβ42 dimer formation.

## Graphical abstract

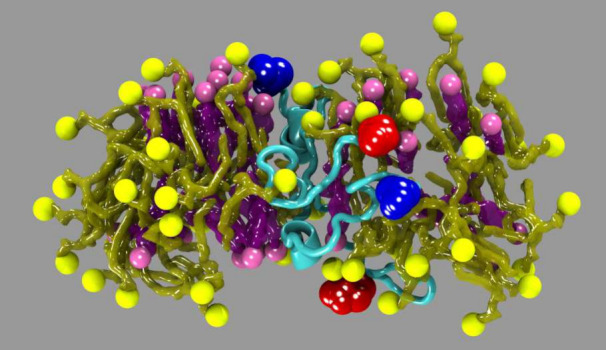

## 1 Introduction

Amyloid β-protein (Aβ) plays a seminal role in Alzheimer’s disease (AD), the leading cause of dementia worldwide.^1^ AD is characterized by extracellular plaques containing 40 and 42 residues-long Aβ40 and Aβ42, respectively, intracellular neurofibrillary tangles, and neuronal loss. Amyloid cascade hypothesis posits that AD is triggered by aberrant Aβ assembly and in its revised form identifies soluble Aβ oligomers as the key toxic species.^2–5^ Aβ40 and Aβ42 are the most abundant variants produced in the brain, whereby Aβ42 correlates with the severity of the disease and is generally considered to be more toxic than Aβ40.

As Aβ is produced throughout a life span, Aβ monomers are unlikely to be toxic– they may even be neuroprotective.^6–8^ In solution, Aβ monomers typically coexist with Aβ oligomers of different sizes, making it challenging to discern at which assembly stage Aβ gains its toxic function. There is no agreed-upon experimental protocol that produces a unique toxic oligomer species.^9^ While dimers have been reported as the smallest toxic species, oligomers as large as 32-mers, and larger protofibrils have also been shown to be toxic.^10–16^ There is no consensus on which Aβ oligomer species or conformation mediates toxicity.^17^ Substantial evidence shows that Aβ oligomers interact with cellular membranes and disrupt calcium homeostasis, which leads to cell dysfunction.^14,18–24^ Proposed mechanisms of membrane disruption include Aβ oligomers that (i) carpet the outer leaflet of a membrane, (ii) get inserted into a bilayer by forming ion channel permeable to calcium ions, and (iii) extract lipids from a bilayer via a detergent effect.^25^ The question of why Aβ40 and Aβ42 monomers are not toxic was addressed by Maiti and collaborators who thermodynamically stabilized Aβ40 and Aβ42 monomers at submicromolar concentrations,^26^ and demonstrated that these species possess much lower affinity for lipid membranes than soluble Aβ40 or Aβ42 comprising a mixture of monomers and oligomers up to decamers.^27^ Interactions of Aβ with membranes are critical for understanding mechanisms and pathways of Aβ oligomer-mediated membrane disruption that leads to AD pathology.^28–30^ It is currently unclear why Aβ oligomers interact with a membrane more avidly than monomers, and how these interactions lead to a membrane disruption in AD.

Molecular dynamics (MD) offers a unique set of tools for unraveling atomistic details of early stages of Aβ folding and assembly in solution as well as Aβ-lipid interactions. Several fully atomistic MD studies examined folding and assembly of full-length Aβ42 in explicit solvent using either enhanced sampling techniques or multiple replica MD trajectories to capture intrinsically disordered nature and resulting conformational polymorphism.^31–36^ While the details of Aβ42 conformational ensembles depend on the MD force field used in simulations, most studies report that Aβ42 monomers are dominated by a statistical coil, consistent with its intrinsically disordered nature. A number of MD studies have also explored Aβ42 interactions with lipid bilayers, whereby Aβ42 monomer or oligomer is initially placed into a proximity of or inserted into a preformed lipid bilayer, followed by the analysis of time evolution of Aβ-lipid bilayer interactions.^37–44^ Davis and Berkowitz reported that Aβ42 monomer adheres to zwitterionic dipalmitoylphosphatidylcholine (DPPC) or anionic dioleoylphosphatidylserine (DOPS) lipid bilayers, but does not exhibit significant changes in its secondary structure.^37,38^ Using united-atom MD simulations, Brown and Bevan examined Aβ42 tetramer formation and interactions of tetramers with preformed lipid bilayers of two compositions, pure 1-palmitoyl-2-oleoyl-*sn*-glycero-3-phosphocholine (POPC) bilayers and cholesterol (CHOL)-rich lipid rafts.^39^ Whereas Aβ42 tetramers did not penetrate lipid bilayers, positively charged Aβ42 residues interacted predominantly with lipid head groups, which perturbed the pure POPC lipid bilayer more than the raft-containing bilayer.^39^ Fatafta and collaborators reported that Aβ42 dimers in aqueous solution convert from disordered coils to β-sheet-rich conformations unlike in the presence of a neuronal membrane-mimicking lipid bilayer comprising POPC, 1-palmitoyl-2-oleoyl-*sn*-glycero-3-phosphoethanolamine (POPE), 1-palmitoyl-2-oleoyl-*sn*-glycero-3-phospho-l-serine (POPS), sphingomyelin (SM), and monosialotetrahexosylganglioside (GM1), where the β-sheet content in Aβ42 dimers is suppressed due to preferential interactions of dimers with GM1.^41^ In a follow up study, Fatafta et al. explored the lipid-chaperone hypothesis of membrane disruption^45^ by allowing Aβ42 monomer to form stable complexes with a single or three POPC or DPPC lipids.^42^ The interaction of selected Aβ42-lipid complexes with lipid bilayer was then examined through microsecond-long MD.^42^ Aβ42 monomer in a complex with three lipids exhibited a deeper adsorption into the bilayer but did not penetrate the bilayer.^42^ Replica-exchange MD studies by Lockhart and Klimov examined interactions of Aβ(10 – 40) monomer with preassembled dimyristoylphosphatidylcholine (DMPC) lipid bilayer and found that the peptide penetrated the bilayer core.^46^ Aβ(10 – 40) monomer insertion was, however, shallow and caused an indentation of a bilayer and only minor structural perturbations in lipid tail conformations.^47^ The addition of CHOL to the DMPC bilayer resulted in a partial expulsion of Aβ(10 – 40) monomer from the hydrophobic tail region of the bilayer.^48^ Feng and collaborators reported on damaging effects of Aβ42 dimer on the DPPC lipid bilayer, whereby each of the four Aβ42 dimers were inserted into the lipid bilayer prior to the MD simulations.^40^ Similarly, Press-Sandler and Miller studied conformational dynamics of four Aβ42 dimers, which were initially preinserted into a DOPC lipid bilayer.^44^ Wang and Guo explored the effect of different CHOL concentrations on the stability of Aβ42 dimers, which were initially inserted into a preformed CHOL-containing lipid bilayers.^43^ While Xiang and collaborators observed a spontaneous insertion of Aβ(11 – 42) dimers through pentamers into a preformed bilayer comprising POPC and CHOL, the peptides under investigation were not full-length Aβ42 peptides.^49^ Mechanical properties of a POPC bilayer with preinserted oligomers of N-terminally truncated Aβ(11 – 42) were also investigated by Grasso and collaborators.^50^ To the best of our knowledge, no MD study to date reported a spontaneous insertion of Aβ42 monomer, Aβ42-lipid complex, or Aβ42 dimer into a lipid bilayer.

Considering the amphiphilic nature of Aβ42, it is reasonable to ask how many lipids does it take to encapsulate the C-terminal hydrophobic regions of Aβ42. Andrews and collaborators investigated how the presence of physiological levels of salt and free DMPC lipids modulate the conformational dynamics of Aβ42 monomers.^51^ In this study, three different systems were explored: Aβ42 monomers alone, Aβ42 monomers with 12 DMPC lipids, and Aβ42 monomers with 48 DMPC lipids, each in the absence and presence of salt, using ten replica MD trajectories per system. Surprisingly, in all trajectories, Aβ42 monomer coassembled with free DMPC lipids to form a stable disordered complex, whereby Aβ42 monomer adhered to the surface of the DMPC cluster.^51^ Aβ42 monomer did not get encapsulated by DMPC lipids at any condition, suggesting that Aβ42 monomers do not interact with DMPC lipids in a way that would disrupt lipid–lipid assembly.^51^

Experimental and computational studies have revealed that lipid composition strongly affects Aβ-membrane interactions. CHOL-rich lipid rafts play a central role in Aβ biogenesis^52^ as well as Aβ oligomer-induced membrane disruption.^28,53–55^ Some studies reported that GM1 gangliosides promote Aβ-lipid interactions.^56,57^ Williams and collaborators optimized the lipid composition of large unilamellar vesicles to include 68% DMPC, 30% CHOL, and 2% GM1 for experimental studies of Aβ42-induced membrane damage.^58,59^

We here hypothesize that Aβ42 monomers and dimers interact with lipids in distinct ways, revealing plausible mechanisms through which dimers but not monomers perturb lipid bilayer and thus trigger AD. To test this hypothesis, we perform extensive MD simulations. In contrast to MD studies of Aβ42-lipid interactions reported in the literature, we set up the initial state by using spatially separated, randomly placed lipids and examine how these free lipids assemble into a lipid bilayer in the absence and presence of Aβ42 monomers or dimers, thereby mimicking *in vitro* experiments by Quist and collaborators, in which association of several amyloidogenic proteins with free lipids resulted in formation of ion-channel-like structures.^19^ Findings of this study support our hypothesis and provide unique insights into stochastic mechanisms of lipid bilayer disruption that are mediated by Aβ42 dimers but not monomers.

## 2 Results

We examine the effect of lipids on Aβ42 monomers and dimers and *vice versa* by performing MD simulations of five distinct systems: (i) 100 lipids, Aβ42 monomers in the (ii) absence and (iii) presence of 100 lipids, and Aβ42 dimers in the (iv) absence and (v) presence of 100 lipids (see Methods for details). Systems of lipids without Aβ42 (i), Aβ monomers without lipids (ii), and (iv) Aβ42 dimers without lipids are control systems that allow for studying the effect of lipids on Aβ42 monomers and dimers as well as the effect of Aβ42 monomers and dimers on lipid assembly. Initially, lipids are randomly placed into the simulation box for systems (i), (iii), and (v). We use the lipid composition of 68% DMPC, 30% CHOL, and 2% GM1, which was reported to be sensitive to Aβ42 oligomer-induced damage imparted on large unilamellar vesicles (LUVs), resulting in leakage of a fluorescent dye from the interior of LUVs.^58,59^ Note that only 2 out of 100 lipids are GM1 molecules, which are included exclusively to mimic the lipid composition and not to draw any conclusions on the effect of GM1 on lipid assembly in the absence or presence of Aβ42.

As an intrinsically disordered protein (IDP), Aβ42 is notoriously difficult to study due to a large ensemble of conformations it can adopt in aqueous solution.^34,51,60^ To enhance the sampling of the phase space and explore multiple assembly pathways, we acquire ten replica trajectories, each 0.5 μs-long, for each system (i-v). Convergence of MD trajectories is addressed in Supporting Information. All Aβ42-containing systems (ii-v) are characterized by significant trajectory-to-trajectory variability, which can be noted in time evolution of (a) RMSD values of backbone atoms (Figure S1), (b) end-to-end distance values between the N-terminal and C-terminal residues (N–C distance) of Aβ42 (Figure S2), and (c) RMSF values (Figure S3). Snapshots of Aβ42 monomer and dimer conformations in the absence and presence of lipids at 500 ns are displayed in Figure S4.

Time evolution of representative trajectories of the five systems are shown in Figure 1. System (i) forms hydrophobically collapsed lipid micelles within the first 100 ns, which then merge into a single lipid bilayer-like assembly within 300 ns–500 ns (Figure 1A). In system (ii), Aβ42 monomer in the absence of lipids samples an ensemble of partially unfolded conformations (Figure 1B). System (iii) shows Aβ42 monomer adjacent to one lipid micelle after the initial hydrophobic collapse at 100 ns, followed by merging of micelles into a lipid bilayer-like structure with Aβ42 monomer residing at the side of the bilayer with exposed lipid tail groups (Figure 1C). Aβ42 dimer in the absence of lipids, representative of system (iv), does not dissociate during 500 ns (Figure 1D). Time evolution of Aβ42 dimer associating with lipids, system (v), also shows a hydrophobic collapse into lipid micelles, whereby the dimer remains adjacent to one lipid micelle at 100 ns and later to the lipid bilayer-like cluster (Figure 1E).

**Figure 1:**
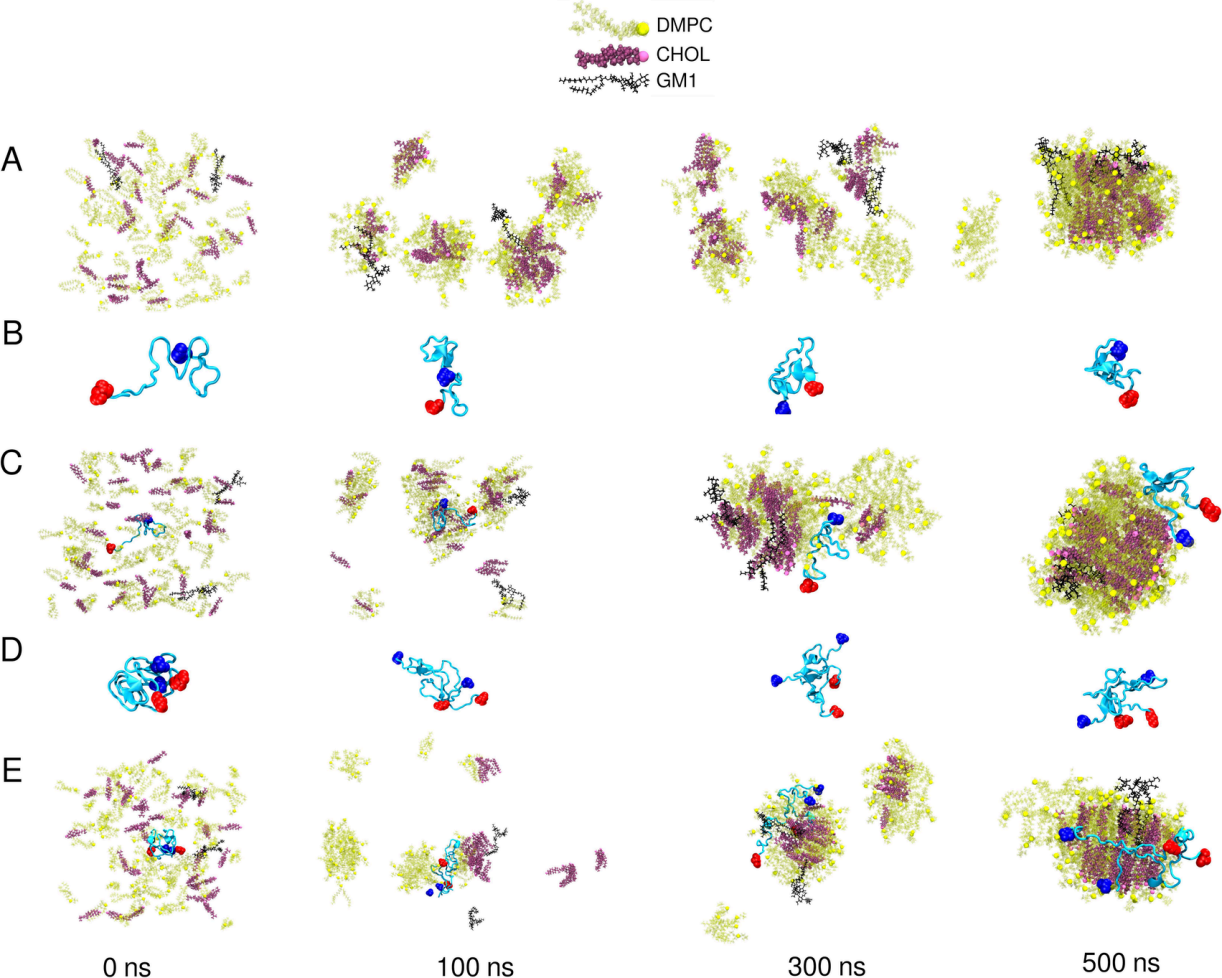
Time evolution of assembly of five distinct systems under study: (A) lipids comprising 68 DMPC, 30 CHOL, and 2 GM1 molecules, Aβ42 monomer (B) in the absence and (C) in the presence of lipids, Aβ42 dimer (D) in the absence and (E) in the presence of lipids. Initially, all lipids are randomly placed in the simulation box. Snapshots of representative MD trajectories, i.e. one of ten replica trajectories per system, are shown at 0, 100, 300, and 500 ns of simulations. 68 DMPC, 30 CHOL, and 2 GM1 molecules are displayed in yellow, purple, and black, respectively. Aβ42 is shown in cyan with N-terminal D and C-terminal A displayed as red and blue spheres, respectively. Images were created using Visual Molecular Dynamics (VMD) software package.^61^

The primary structure of Aβ42 is D_1_AEFRHDSGY­EVHHQKLV­FFAEDVGS­NKGAII­GLMVG­GVVIA_42_. Specific peptide regions along the sequence: L_17_VFFA_21_, I_31_IIGLMV_36_, and V_39_VIA_42_ are hereafter referred to as the central hydrophobic cluster (CHC), midhydrophobic region (MHR), and the C-terminal region (CTR), respectively.

### 2.1 Lipids Destabilize Aβ42 Dimers

To examine Aβ42 dimer stability, we monitor a minimal distance between the two peptides in a dimer along each MD trajectory of systems (iv) and (v) (Figure 2, panels A-B). Using a minimal interpeptide distance of 0.5 nm to define a dimer (see Methods), Figure 2, panels A-B shows that in the absence of lipids only 1 out of 10 dimers dissociates into two monomers (Figure 2A). In the presence of lipids, however, 4 out of 10 dimers (trajectories 2, 6, 7, and 8) dissociate into two monomers (Figure 2B). Thus, Aβ42 dimers are four times more likely to dissociate into monomers in the presence of lipids. We then calculate the average number of interpeptide contacts in Aβ42 dimers by averaging time frames within 400–500 ns, separated by 1 ns, of each trajectory. Figure 2C–D shows a large trajectory-to-trajectory variability in the average number of interpeptide contacts. Nonetheless, the final ensemble averages and SEM values for dimers in absence and presence of lipids, 47 ± 0.69 and 27.1 ± 0.67, respectively, indicate a statistically significant difference. The above SEM values are calculated by using statistics over time frames of all ten trajectories. Even if the SEM values reflected only trajectory-to-trajectory variability, resulting in a 10-fold increase in the error bars, the difference in the number of interpeptide contacts in dimers in the absence and presence of lipids would still be significant. Notably, trajectories 2, 6, 7, and 8 of system (v), corresponding to dissociated dimers, show the lowest number of interpeptide contacts (Figure 2C–D). Thus, on the observed time scale of 500 ns, lipids destabilize Aβ42 dimers by significantly reducing the number of quaternary contacts. Note that the observed Aβ42 dimer dissociation may correspond to a reorganization step that would eventually lead to different dimer conformations with increased stability in the presence of lipids.

**Figure 2:**
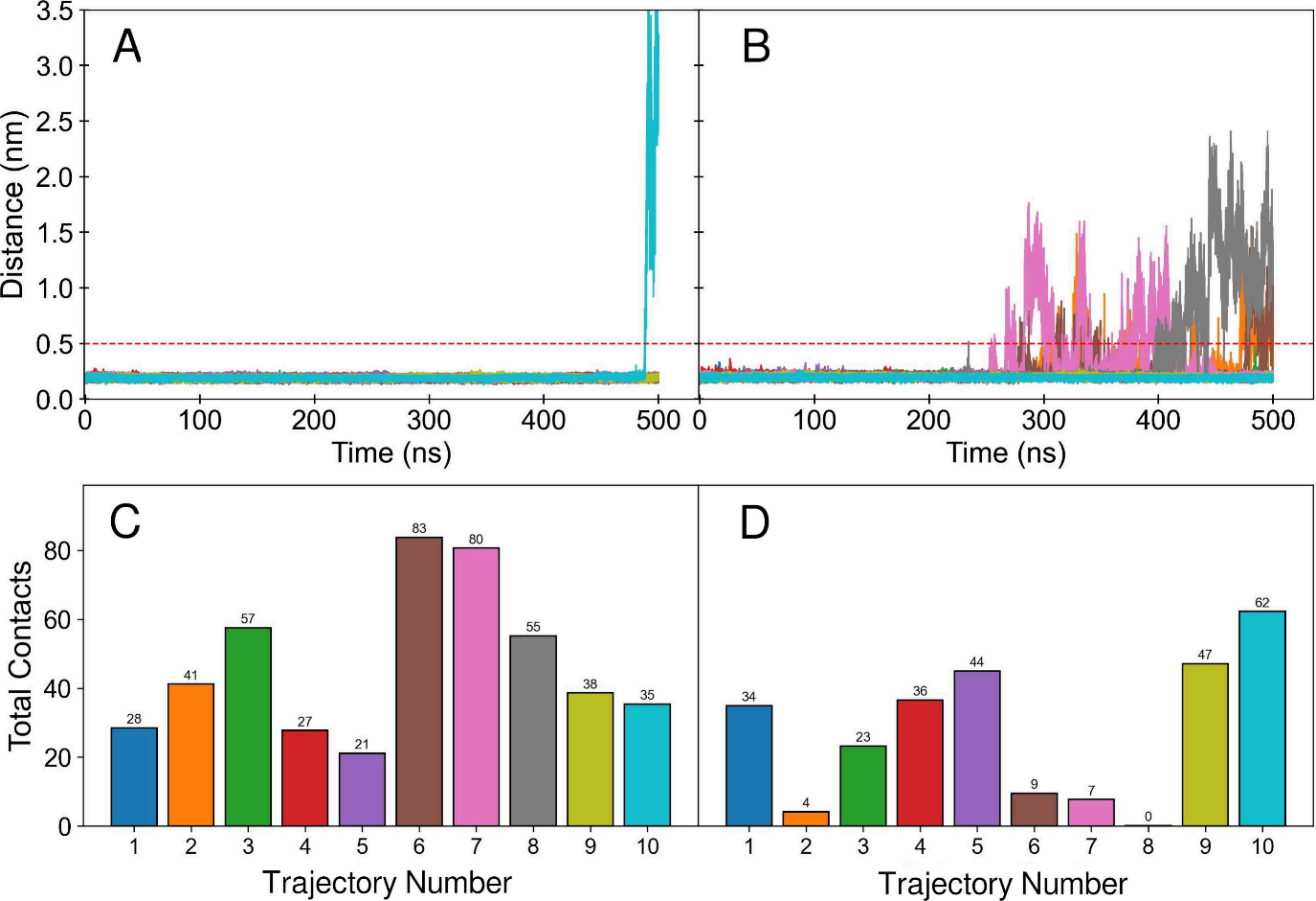
(A, B) The minimum interpeptide distance in the Aβ42 dimers in the (A) absence and (B) presence of lipids versus simulation time. Minimum interpeptide distances for ten replica trajectories per system are displayed in distinct colors. The red dashed line corresponds to the minimal interpeptide distance of 0.5 nm, which is used in the definition of a dimer (see Methods). (C, D) The average number of interpeptide contacts in a Aβ42 dimer conformation for each of the ten replica trajectories in the (C) absence and (D) presence of lipids.

### 2.2 Lipids Reduce Structural Disorder in Aβ42 Monomers but Not in Aβ42 Dimers

Visual inspection of Figure 1 (C, E) shows that both Aβ42 monomers and dimers avidly interact with lipids. We here examine to what extent Aβ42-lipid interactions affect the secondary structure of Aβ42 monomers and dimers. The secondary structure (coil, β-strand, turn, and helix) is analyzed in two ways, by calculating the average secondary structure content per peptide and the average per-residue secondary structure propensities (see Methods). In monomers, the presence of lipids significantly decreases the average coil content while both the average β-strand and turn content increase, suggesting that lipids promote more ordered Aβ42 monomer conformations (Figure 3A). A minor decrease in the helical content is observed for monomers in the presence of lipids (Figure 3A). The average secondary structure content in Aβ42 dimers is less affected by lipids than in monomers (Figure 3B). In Aβ42 dimers, lipids slightly increase the average coil and turn content, while decreasing the average β-strand and helical content. Thus, although lipids reduce Aβ42 dimer stability, they do not strongly affect its average secondary structure content. A comparison of the secondary structure content between Aβ42 monomers and dimers in Figure 3 (C,D) shows that in the absence of lipids, dimers are more structured than monomers as reflected in lower coil content per peptide in dimers, whereas β-strand, turn, and helical content contributions are higher than in monomers (Figure 3C). In contrast, in the presence of lipids, dimers are characterized on average by a lower β-strand and turn than monomers (Figure 3D). Both in the absence and presence of lipids, dimers have a higher helical content than monomers although the overall contribution from all types of helical structure does not exceed 0.05. The effect of lipids on Aβ42 per-residue secondary structure propensities is described on pages S1–S2 of Supporting Information and shown in Figures S5 and S6, whereas tertiary and quaternary structure changes due to the presence of lipids are described on pages S2–S3 of Supporting Information and shown in Figure S7. In the presence of lipids, Aβ42 monomers gain secondary and tertiary structure, whereas Aβ42 dimers lose tertiary and quaternary structure without significant changes in the secondary structure.

**Figure 3:**
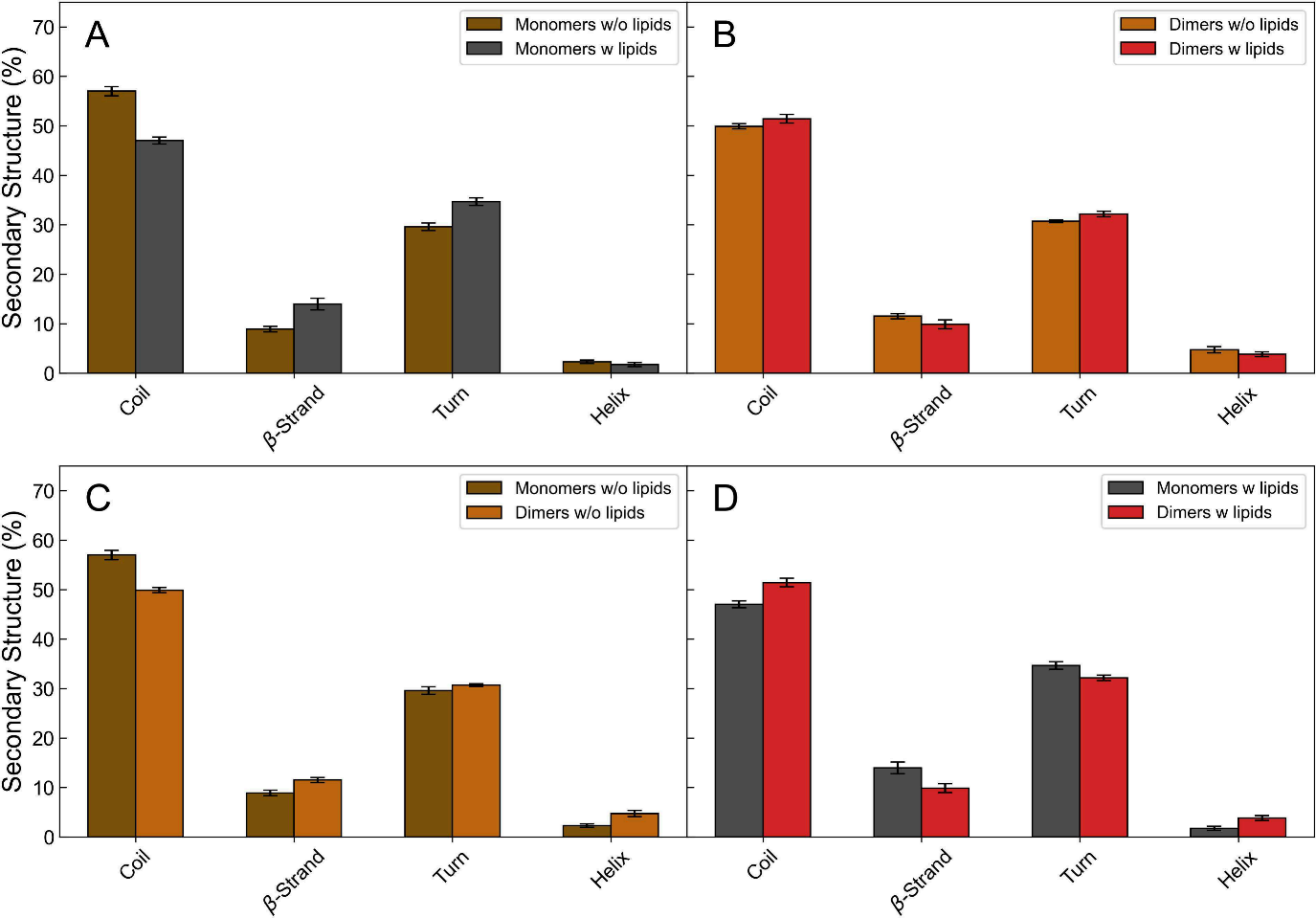
Effect of lipids on the average secondary structure in Aβ42 monomers and dimers. (A) The average coil, strand, turn, and helical content in Aβ42 monomers and dimers in the absence and presence of lipids. (B) Average per-residue coil, strand, turn, and helical propensities in Aβ42 monomers and dimers in the absence and presence of lipids. The same average coil, strand, turn, and helical content of monomers versus dimers is shown in the (C) absence and (D) presence of lipids. The error bars correspond to standard error of the mean (SEM) values.

### 2.3 Aβ42 Dimers Interact with CHOL More Avidly than Aβ42 Monomers

Per-residue contact probabilities between Aβ42 monomers/dimers and lipid molecules (DMPC or CHOL) are calculated as described in Methods. Per-residue contact probabilities of Aβ42 monomers with DMPC are on average higher than the respective probabilities with CHOL (Figure 4, panels A-B). Monomers thus interact more avidly with DMPC than with CHOL, likely because there are more than twice as many DMPC than CHOL molecules available for interactions. Specific residues in Aβ42 monomers with the highest DMPC contact probabilities are, in ranking order: F4, F19, L17, I32, I31, Y10, and V12, i.e., mostly hydrophobic residues. Similarly, hydrophobic residues F4, I31, and L34 in monomers form the most frequent contacts with CHOL. Aβ42 dimers form, on average, more contacts with DMPC than with CHOL (Figure 4, panels C–D). Dimers exhibit parable or slightly higher number of contacts with DMPC than monomers. Notably, dimers make on average more contacts with CHOL than monomers. This unexpected result indicates that each peptide in Aβ42 dimers interacts with CHOL more avidly than Aβ42 monomers. Residues I32, F4, F19, and L17 in dimers are associated with the highest DMPC contact probabilities. The most probable contacts between dimers and CHOL are formed by residues I32 and L17. In dimers, I32-CHOL and L17-CHOL contact probabilities are higher than I32-DMPC and L17-DMPC contact probabilities, indicating a strong affinity of I32 and L17 in dimers for interacting with CHOL. Figure 4E-F displays dimer-to-monomer differences in DMPC and CHOL contact probabilities, respectively. Dimers interact with DMPC more than monomers through their N-terminal residues up to H13 and residues in region I32-M35. In addition, dimers interact with CHOL more than monomers along most of the sequence.

**Figure 4:**
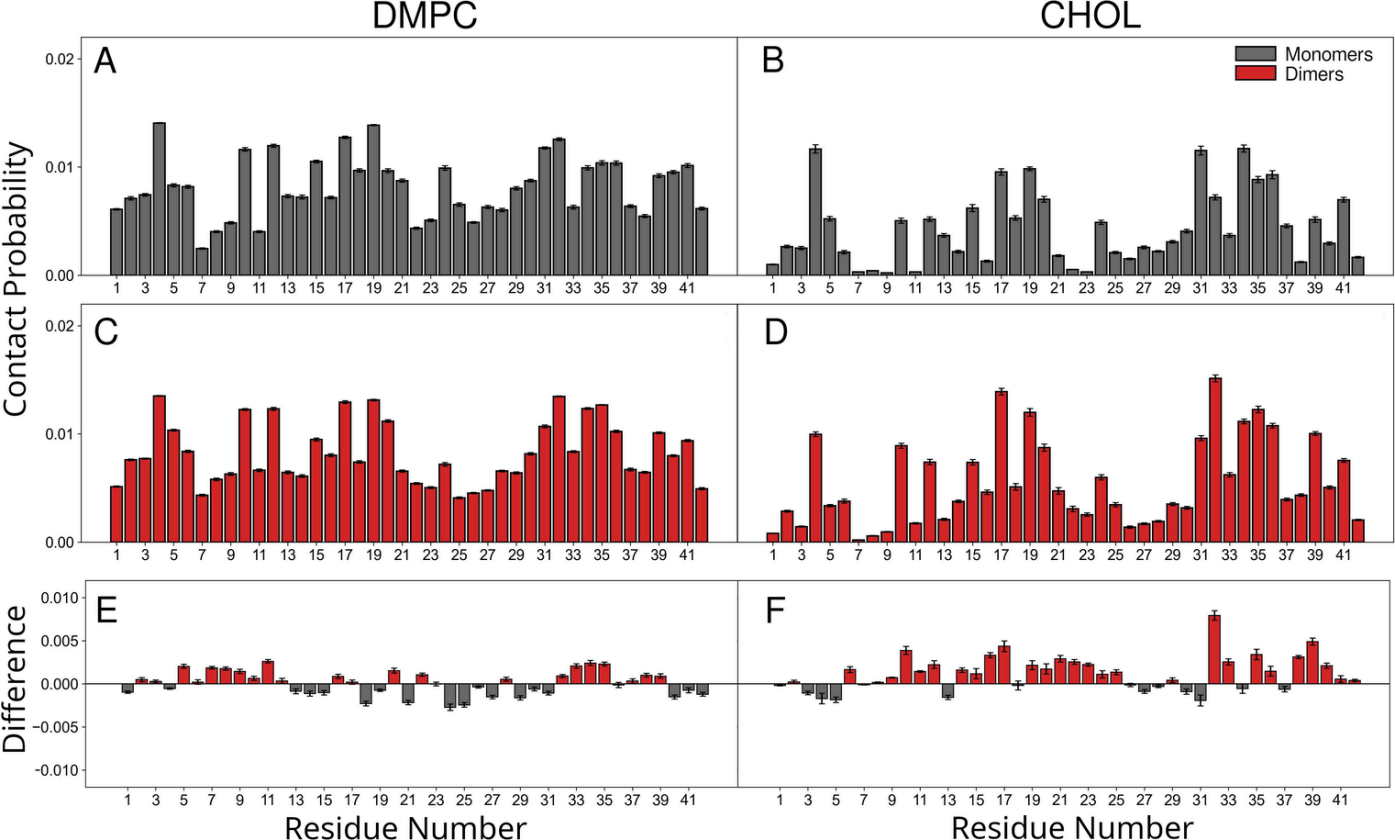
(A, C, E) Contact probability between Aβ42 and DMPC. (B, D, F) Contact probability between Aβ42 and CHOL. Per-residue contact probabilities between Aβ42 (A, B) monomers or (C, D) dimers with DMPC (A and C) or CHOL (B and D). Panels E and F show the dimer minus monomer differences in per-residue contact probabilities of Aβ42 with DMPC and CHOL, respectively, to elucidate the residue-level changes due to Aβ42 assembly state. (A–F) The error bars correspond to the SEM values.

We next asked whether the most probable Aβ42-lipid contacts shown in Figure 4 are a result of prolonged contacts between specific amino acid residues and DMPC or CHOL molecules. To this end, we calculated average per-residue residence times of contacts with DMPC and CHOL lipids as described in Methods. Figure S8 shows that none of the residence times is longer than 150 ps, indicating that Aβ42 contacts are frequent and short-lived. The residence times for contacts with DMPC lipids are overall longer than for contacts with CHOL lipids, likely because DMPC lipids are more than twice as abundant than CHOL lipids. Aβ42 residues D1 and R5 in contact with DMPC exhibit the longest residence times. In addition, DMPC contacts with S26 in monomers and with N27 and G33 in dimers are also longer than for most other residues. Aβ42 contacts with CHOL show less residue-to-residue variability. In dimers, contacts with CHOL are longer than in monomers for residues in the N-terminal and C-terminal regions, whereas in monomers, residues F20-A30 are in contact with CHOL longer than in dimers. The regression analysis between the average per-residue residence times and contact probabilities reveals weak to moderate correlations. In the case of contacts with DMPC, the correlation coefficients are comparable, −0.45 and −0.37 for monomers and dimers, respectively. In the case of contacts with CHOL, the correlation coefficients are 0.11 and 0.44 for monomers and dimers, respectively. The increase in the correlation coefficient for CHOL in dimers relative to monomers is interesting and consistent with dimers displaying more frequent contacts with CHOL than monomers.

### 2.4 Lipids Assemble into a Bilayer with a CHOL-Rich Domain

Time evolution of representative trajectories corresponding to systems (i), (iii), and (v) in Figure 1 (panels A, C, E) shows that lipids in the absence or presence of Aβ42 cluster together. The analysis of cluster sizes performed on ten replica MD trajectories per system for systems (i), (iii), and (v) indicates that in most trajectories, lipids form a single cluster within 400–500 ns (Figure S9). In the lipids-only system (i), lipids assemble into a single cluster in 8 out of 10 trajectories, and in the remaining 2 trajectories, lipids form two clusters (Figure S9, panel A, trajectories 3 and 4). In the system of lipids coassembled with Aβ42 monomer, a single cluster of lipids and Aβ42 forms in 9 out of 10 trajectories; in the remaining trajectory, Aβ42 monomer and lipids form two clusters within 400–500 ns (Figure S9, panel B, trajectory 5). All 10 trajectories in the system of lipids coassembling with Aβ42 dimers are characterized by a single cluster within 400–500 ns. Thus, lipids cluster together and the presence of Aβ42 promotes lipid assembly. Figure S10 further shows that DMPC lipids also form a single cluster except for trajectories 3 and 4 of system (i) and trajectory 5 of system (iii). Importantly, although less abundant than DMPC, CHOL in systems (i), (iii), and (v) exhibits a high propensity to cluster together, forming a single domain within the lipid cluster (Figure S10). Thus, DMPC and CHOL molecules within the lipid cluster are not uniformly distributed but rather form DMPC-rich and CHOL-rich domains.

To gain insight into the time scales of lipid assembly, Figure S11 shows time evolution of lipid cluster size distributions for systems of 100 lipids (i) alone, (iii) with Aβ42 monomers, and (v) with Aβ42 dimers. In the absence of Aβ42, the lipid assembly is the slowest, whereby trajectories 3 and 4 contain 2 clusters even within 400–500 ns. The presence of Aβ42 monomer appears to speed up this process, leaving only trajectory 5 with 2 clusters within 400–500 ns. In the presence of Aβ42 dimers, a single lipid cluster is observed for all 10 replica trajectories within 450–500 ns. Trajectory 1 of system (v) forms 2 lipid clusters at 400 ns. It is important to note that all 10 trajectories of this system with Aβ42 dimers form a single cluster within 400–500 ns if Aβ42 is included in the definition of a cluster, consistent with the results shown in Figure S9. Thus, results in Figure S11 demonstrate that lipids assemble into a single cluster faster in the presence of Aβ42.

Visual inspection of MD trajectories suggests that lipids organize themselves into lipid bilayer-like structures. Notably, these structures are not lipid bilayers with clear-cut boundaries akin to those obtained in MD simulations of preassembled lipid bilayers with a fixed normal to the bilayer where the lateral solvent exposure of lipid tails is avoided through periodic boundary conditions. Instead, the bilayer-like structures observed in our simulations are completely immersed in the solvent and exhibit round micelle-like edges with lipid head groups exposed to the solvent to minimize the solvent exposure of hydrophobic lipid tail groups. To quantify the underlying order of the observed assemblies, we monitor time evolution of the DMPC and CHOL order parameters along each trajectory of lipid-rich systems (i), (iii), and (v), as described in Methods. Figure S12 shows that for most trajectories, nematic DMPC and CHOL order parameters reach steady state values by 400 ns of simulations.

We then calculate density profiles of head groups of DMPC and CHOL (see Methods). Figure 5 (panels A-F) shows density profiles of DMPC and CHOL for ten replica trajectories per system (thin lines) alongside their ensemble averages (thick solid lines). Trajectories 3 and 4 from system (i) and trajectory 5 from system (iii) are excluded from ensemble averages because lipids (without and with Aβ42 monomer) in these three trajectories do not form a single cluster within 400–500 ns. Indeed, density profiles of these excluded trajectories strongly deviate from the density profiles of the other trajectories (Figure 5, panels A-D, thin dashed lines). The ensemble averages of DMPC and CHOL density profiles reveal two peaks corresponding to average locations of the head groups, indicative of a bilayer organization. Notably, the density profiles for CHOL are significantly sharper with higher maxima and a lower minimum flanked between the two maxima (Figure 5, panels B, D, F) than the density profiles for DMPC (Figure 5, panels A, C, E). This result indicates that within a bilayer, CHOL is significantly more ordered than DMPC. The average DMPC and CHOL density profiles of the three systems (lipids-only, lipids with Aβ42 monomer and lipids with Aβ42 dimer) are displayed in Figure 5, panels E and F, respectively. Although DMPC and CHOL density profiles of the three systems nearly overlap, both density profiles get slightly enhanced in the presence of Aβ42 monomers and dimers.

**Figure 5:**
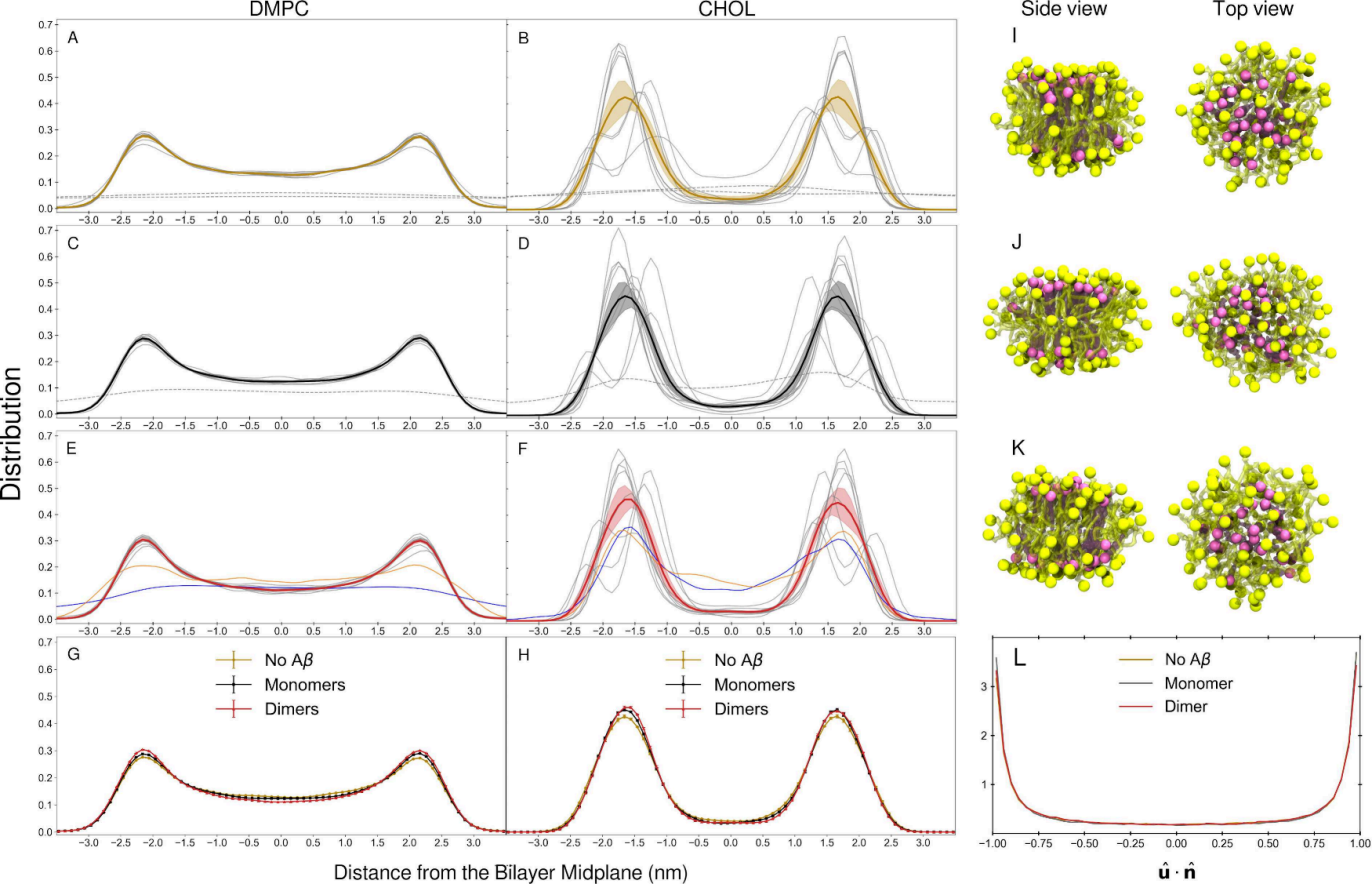
Lipid headgroup density profiles along the (A, C, E) DMPC director (N-headgroup) and (B, D, F) CHOL director (O-group) under three conditions: (A, B) in the absence of Aβ42, in the presence of (C, D) Aβ42 monomers and (E, F) Aβ42 dimers. Thin lines represent individual trajectory profiles. Two thin dashed lines in A and B corresponding to trajectories 3 and 4 (see panel A in Figure S9) and a single thin dashed line in C and D corresponding to trajectory 5 (see panel B in Figure S9) are excluded from the averaging because these systems are not fully assembled into a single cluster within 400–500 ns. Blue and orange lines in (E, F) correspond to trajectories 1 and 2, respectively. Bold lines in (A–F) represent the average lipid density profiles. (G, H) Comparison of the average lipid density profiles for all three conditions with error bars representing the SEM values. (I–K) Side and top views of a typical lipid bilayer at 500 ns of simulations for systems (i), (iii), and (v). GM1 and Aβ42 conformations are not displayed for clarity. DMPC and CHOL molecules are colored in green and pink, respectively. Images are created using VMD.^61^ (L) The distribution of projections of all DMPC and CHOL molecular directors to the normal of a bilayer.

The average distance between the peaks of the DMPC density profiles in Figure 5, i.e. the DMPC thickness, is 4.27 ± 0.019 nm, 4.29 ± 0.017 nm, and 4.18 ± 0.11 nm for systems (i), (iii), and (v), respectively. Similarly, the CHOL thickness, defined as the average distance between the peaks of the CHOL density profiles in Figure 5, is 3.17 ± 0.17 nm, 3.15 ± 0.13 nm, and 3.15 ± 0.14 nm for systems (i), (iii), and (v), respectively. Thus, the bilayer thickness is not affected by Aβ42 monomers or dimers, but varies within the bilayer whereby the CHOL-rich phase is associated with 25% thinner bilayer than DMPC-rich local phase. Snapshots of lipid bilayer-like conformations obtained for systems (i), (iii), and (v) in Figure 5(I–K), respectively, show side views with two clearly visible leaflets and top views with a CHOL-rich domain in the center of the bilayer surrounded by a DMPC-rich domain. Because of the presence of micelle-like edges of the bilayer-like structures, we then asked to what extent these lipid assemblies deviate from an ideal spherical micelle. Panel L in Figure 5 shows a normalized distribution of the projections of all lipid directors in the assembly onto the normal to the bilayer, calculated as described in Methods, which has two sharp peaks at −1 and 1, corresponding to angles 0° and 180° for the two leaflets of the bilayer. As expected, there is a nonzero contribution from lipid with projections around zero (lipid directors perpendicular to the bilayer normal) because of the micellar edges of the bilayer. Using a criterion that the lipid directors do not deviate from the lipid bilayer normal more than 45° (cos 45° = 0.7071), the corresponding fraction of lipids can be calculated as the area below this distribution within intervals [−1, – 0.7071] and [0.7071, 1]. The fraction of lipids that satisfies this criterion is 0.682 for systems (i) and (v), i.e. for lipids without Aβ42 and for lipids assembled in the presence of Aβ42 dimers, and 0.703 for system (iii) for lipids assembled in the presence of Aβ42 monomers. Thus, in all three systems slightly more than two-thirds of all lipids are oriented as expected for a lipid bilayer. In contrast, if the lipids would form an ideal spherical micelle with all lipid directors pointing radially, the corresponding fractions of lipids with a polar angle of at most 45° would be (1 – cos 45°)/2 = 0.146 or 0.293 (considering both poles), which is about 2.4-fold smaller than this population in our lipid assemblies, indicating that in our lipid assemblies resemble lipid bilayers more than micelles.

We next calculated the nematic and hexatic order parameters, *S*_2_ and |ψ_6_|, of DMPC and CHOL lipids (see Methods for details) in a bilayer for the three lipid-rich systems (i), (iii), and (v). Figure 6 shows that the nematic order parameter of CHOL is about two times higher than the nematic order parameter of DMPC, demonstrating that CHOL is significantly more ordered than DMPC within the bilayer. Figure 6A shows that average nematic order parameters calculated over ten replica trajectories per system, whereas the average nematic order parameter calculation in Figure 6B excludes trajectories 3 and 4 from system (i) and trajectory 5 from system (iii) due to existence of two lipid clusters with two different orientations. Thus, Figure 6B shows the effect of Aβ42 monomers and dimers on DMPC and CHOL order parameters within a bilayer. Monomers and dimers mixed with lipids both increase the nematic DMPC order parameter, however, the monomer-induced increase is somewhat larger than the dimer-induced increase. Interestingly, while the nematic CHOL order parameter increases in the presence of monomers, it decreases in the presence of dimers. These distinct effects of Aβ42 monomers versus dimers on the CHOL order parameter are consistent with the contrasting effects of lipids on the monomer versus dimer structure and an increased number of contacts with CHOL in dimers relative to monomers.

**Figure 6:**
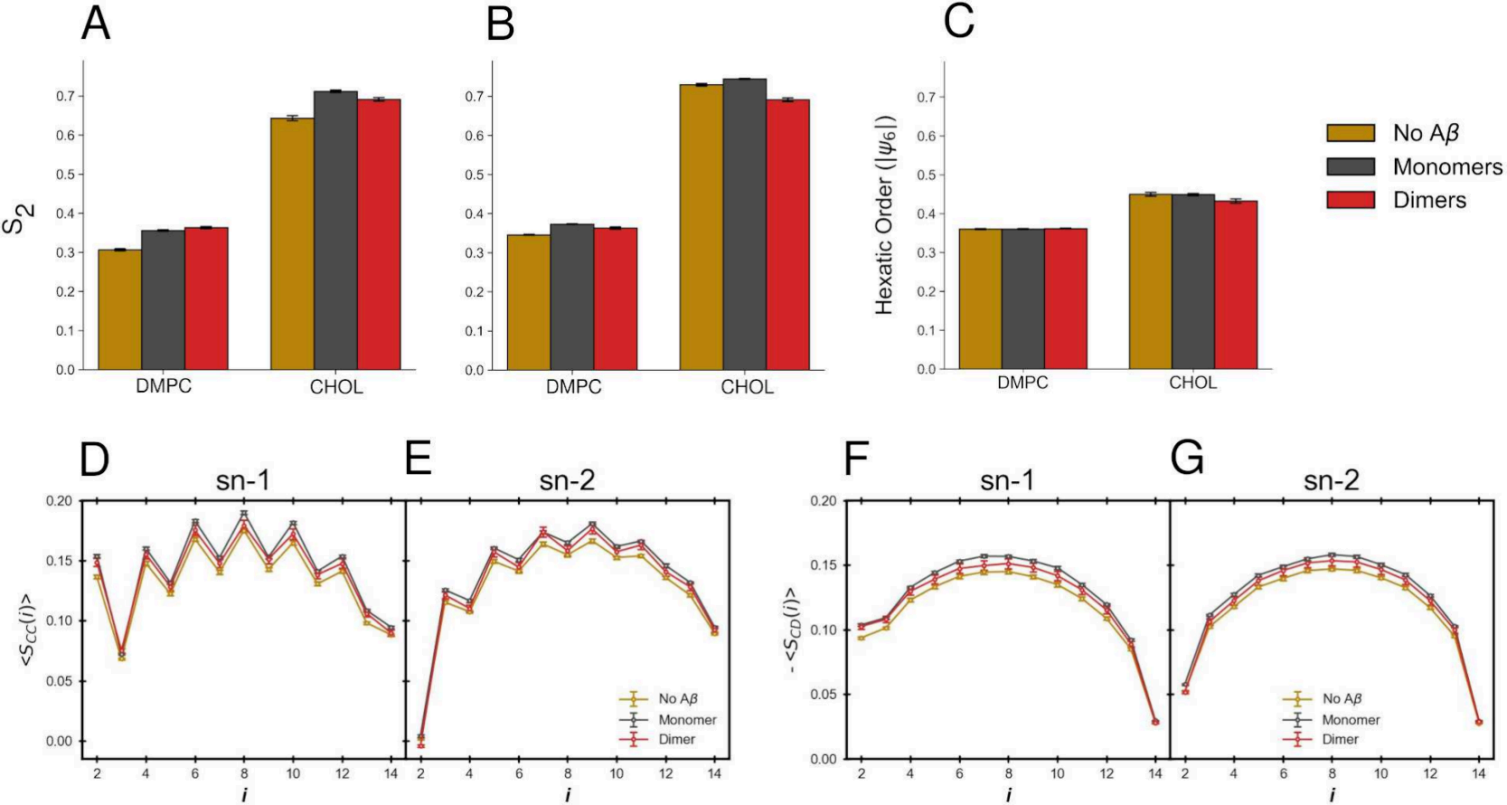
Lipid order parameters for systems (i), (iii), and (v). Nematic DMPC and CHOL order parameter, *S*_2_, calculated by averaging over (A) 10 replica trajectories per system and (B) only trajectories with a single cluster within 400–500 ns. (C) Hexatic DMPC and CHOL order parameters of the lipid bilayer, |Ψ_6_|, calculated by averaging the top- and bottom-leaflet values, using only trajectories with a single cluster within 400–500 ns. (D, E) Carbon–carbon and (F, G) carbon deuterium order parameters of DMPC lipid tails (D, F) sn-1 and (E, G) sn-2, calculated by using only trajectories with a single cluster within 400–500 ns. The error bars correspond to the SEM values.

Hexatic order parameter in the DMPC-rich and CHOL-rich domain of the lipid bilayer and compared in Figure 6C, which indicates that this order parameter is higher in the CHOL-rich domain (0.45) than in DMPC-rich domain (0.35), further indicating that the CHOL-rich domain is more ordered, consistent with a lipid raft-like structure.^62,63^ In contrast to the DMPC hexatic order parameter, which is not affected by the presence of Aβ42, the CHOL hexatic order parameter is slightly lower in the presence of Aβ42 dimers but unaffected by Aβ42 monomers, revealing that dimers but not monomers affect the hexatic ordering in the CHOL-rich domain. Two deuterium order parameters, ⟨*S*_*CC*_⟩ and -⟨*S*_*CD*_⟩, for both DMPC tails, sn-1 and sn-2, which probe the ordering of the carbon–carbon and carbon–hydrogen bonds, respectively, shown in Figure 6(D-G), demonstrate that the presence of Aβ42 dimers and even more so, Aβ42 monomers, increases the DMPC tail order, consistent with the observation that monomers tend to increase the lipid bilayer ordering. A comparison of ⟨*S*_*CC*_⟩ and -⟨*S*_*CD*_⟩ for sn-2 in Figure 6 (panels E and G) to a preassembled lipid bilayer comprising 96 DMPC lipids, reported by Lockhart and Klimov,^47^ shows that these order parameters in our bilayers with micellar edges follows a similar shape, but are by about 30% lower in the absolute values, which is consistent with our estimation that about 30% of DMPC lipids contribute to micellar edges rather than to a bilayer structure. Figure S13 demonstrates significant trajectory-to-trajectory variability of nematic DMPC and CHOL order parameters, which reinforces the need to explore these systems using multiple MD trajectories to capture this diversity.

Snapshots at 500 ns of all MD trajectories of systems (iii) and (iv) are displayed in Figure 7 to illustrate individual Aβ42 monomer and dimer conformations in a complex with lipids. The two GM1 molecules stick out of the bilayer leaflets. While the two GM1 molecules do not self-associate or associate with Aβ42, no reliable conclusions about the role of GM1 can be made due to insufficient sampling.

**Figure 7:**
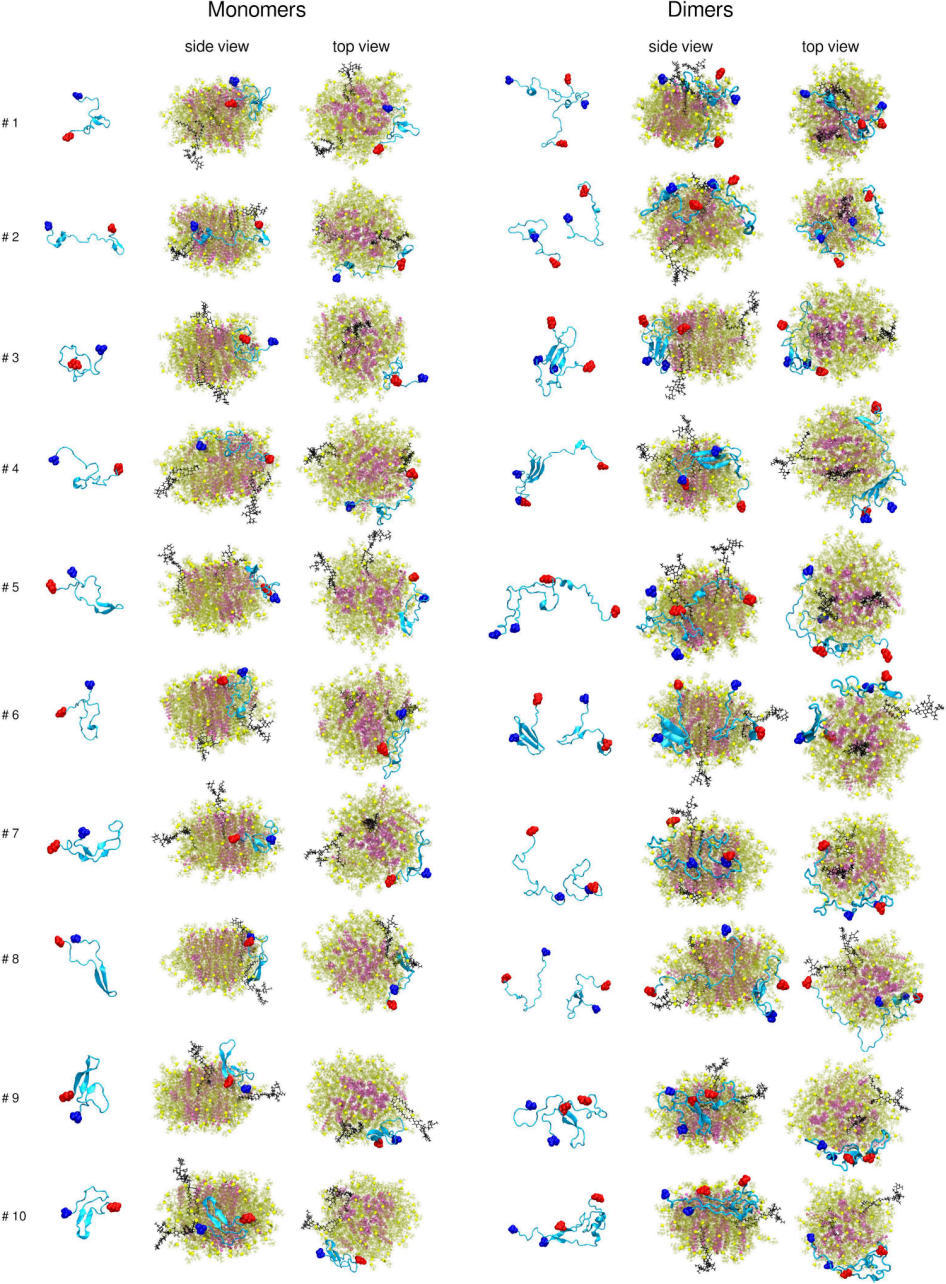
Snapshots of Aβ42 monomers and dimers interacting with lipid bilayer at 500 ns for all 10 replica trajectories per system. DMPC, CHOL, and GM1 molecules are displayed in yellow, purple, and black, respectively. Aβ42 dimer is shown in cyan, with red and blue spheres marking the N- and C-terminal D and A residues, respectively. Images were created using VMD.^61^

### 2.5 Aβ42 Monomer Gets Adhered to a Lipid Bilayer Side

A detailed inspection of time evolution of ten replica trajectories of system (iii) reveals similar assembly pathways across all trajectories. Within the initial 50–100 ns, Aβ42 monomer adheres to one of the lipid micelles during the initial hydrophobic collapse. The lipid micelle with a monomer then grows by merging with other lipid micelles and gets subsequently more ordered until it forms a lipid bilayer by ∼ 400 ns. During the entire assembly process, Aβ42 monomer remains attached to a side of the lipid bilayer with exposed lipid tail groups, as shown in Figure 8A. Aβ42 monomer placement adjacent to the lipid cluster is in line with findings of Andrews and collaborators who reported that 12 or even 48 DMPC lipids coassemble with Aβ42 monomers in a complex, whereby the peptide resides on the surface of the lipid cluster without getting inserted into the cluster.^51^ This placement of Aβ42 monomer adjacent to the lipid bilayer explains why Aβ42 monomer slightly increases nematic DMPC and CHOL lipid order parameters (Figure 6). As shown in the previous subsection, Aβ42 monomer adhered to the side of the lipid bilayer-like structure increases the population of lipids whose directors deviate from the bilayer normal in both directions less than 45°. Thus, Aβ42 monomer partially replaces the population of lipids within the micellar edge of the bilayer, which explains the increase in the nematic, carbon–carbon, and deuterium order parameters.

**Figure 8:**
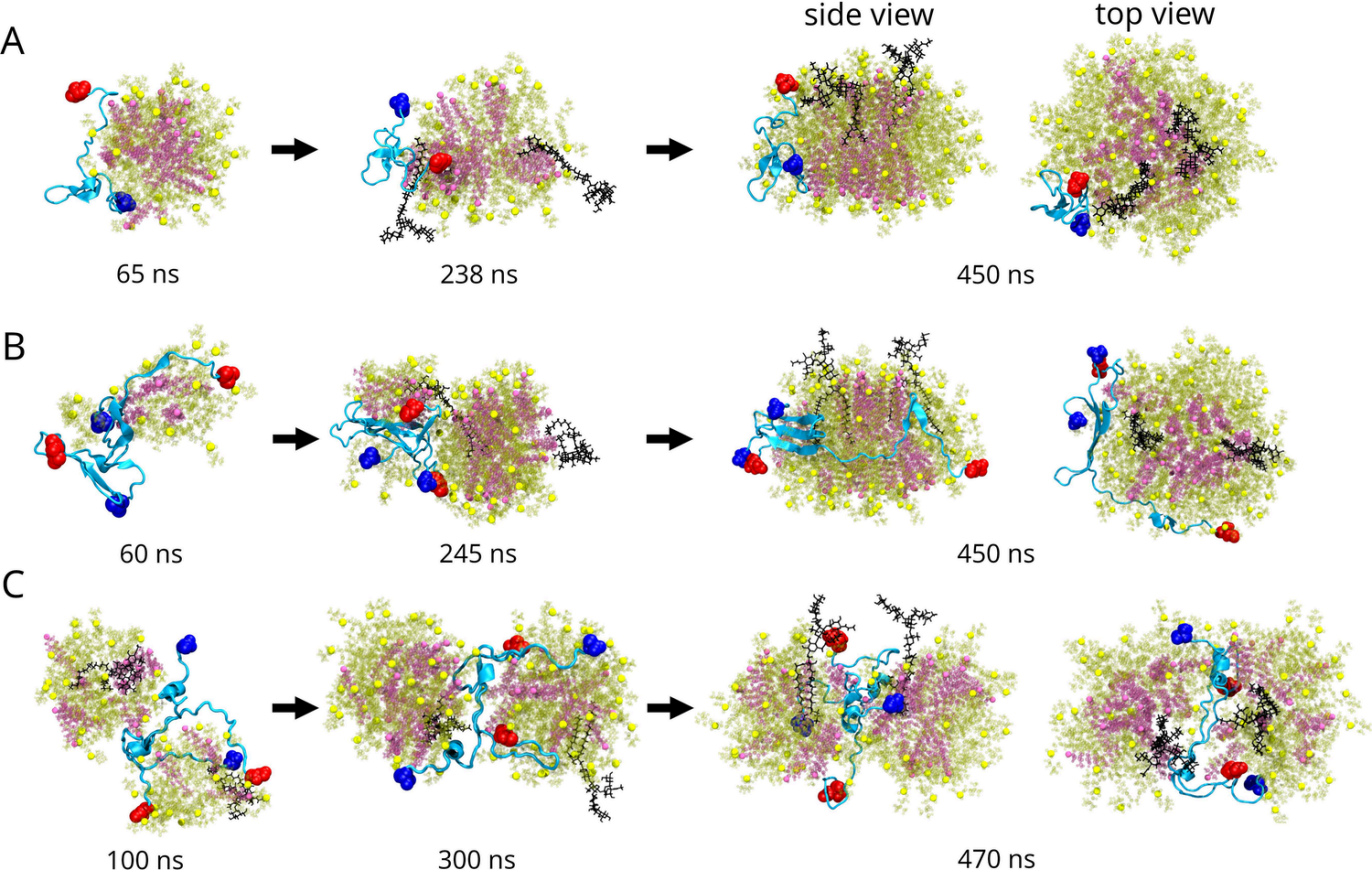
Representative pathways of (A) Aβ42 monomer-lipid assembly with three snapshots from trajectory 1 of system (iii) and (B, C) Aβ42 dimer-lipid assembly with snapshots from trajectories 4 and 1 of system (v), respectively. (A–C) Simulation time is displayed below each snapshot. The third and fourth snapshots of each row show the same configuration from the side and the top of the bilayer, respectively. DMPC, CHOL, and GM1 molecules are displayed in yellow, purple, and black, respectively. Aβ42 is shown in cyan, with red and blue spheres marking the N- and C-terminal D and A residues, respectively. Images were created using VMD.^61^

### 2.6 Multiple Pathways of Aβ42 Dimer Coassembly with Lipids: Adherence, Spontaneous Insertion, and Carpeting

A detailed visual inspection of all 10 trajectories revealed that in contrast to Aβ42 monomers, assembly of Aβ42 dimers with lipids in system (v) is more diverse. The first pathway (trajectories 3, 4, 6, 9, and 10) is similar to the pathway of Aβ42 monomer coassembly with lipids. Within the first 100 ns, Aβ42 dimer associates with one of the lipid micelles, which then grows in size and forms a lipid bilayer, whereby the two peptides of a dimer adhere to one or two sides of the lipid bilayer with solvent exposed lipid tails (Figure 8B). Trajectory 6 is further different from the other trajectories in this group because Aβ42 dimer dissociates into two monomers that remain adhered to two adjacent sides of the bilayer. The second pathway (trajectories 1, 2, 5, 7, and 8), reveals an intermittent insertion of Aβ42 dimer into a lipid cluster, which is not observed for Aβ42 monomers in system (iii). Using a visual inspection, the insertion is defined by an Aβ42 dimer that spans both leaflets of the bilayer. The stability of this insertion, as assessed by the insertion time, varies from trajectory to trajectory. Because time intervals of insertion are challenging to determine by visual inspection alone, time evolution of 11 quantities is monitored for trajectories 1, 2, and 4 (Figure S14). These quantities are (1) the minimal distance between the two peptides in Aβ42 dimer, (2) the solvent accessible surface area (SASA) and (3) the hydrophobic SASA of Aβ42 dimer, the SASA of (4) lipid headgroups and (5) lipid tailgroups, (6) van der Waals and (7) electrostatic energy of Aβ42 dimer-lipid system, and the number of residues per peptide in contact with (8) DMPC tail groups, (9) CHOL tail groups, (10) DMPC head groups, and (11) CHOL head groups. Notably, during insertion events, the number of residues in contact with CHOL tail groups per peptide and the sum of the two energies seem to be anticorrelated, indicating that the insertion maximizes Aβ42 contacts with CHOL tail groups and minimizes the sum of the two energies. Figure S15 shows time evolution of these two quantities for each of the 10 trajectories. Using trajectory 10 as a control without any insertion or carpeting events, the sum of the average and standard deviation is used as a threshold that allows the insertion events to be identified. Red rectangular time intervals within trajectories 1, 2, 5, 7, and 8 correspond to visually identified insertion events longer than 50 ns quite well. The most stable configuration with Aβ42 dimer inserted into a lipid micelle and eventually into a lipid bilayer persists during 70–470 ns of trajectory 1. The corresponding coassembly pathway (Figure 8C) shows Aβ42 dimer in an antiparallel conformation inserted into a lipid micelle at earlier and later times. Similarly, somewhat shorter spontaneous insertion events are observed within time intervals 230–320 ns, 100–220 ns, 110–220 ns, and 30–390 ns of trajectories 2, 5, 7, and 8, respectively. Comparing Figures S15 and 2, we conclude that all insertion events correspond to Aβ42 in a dimeric state. The insertion events in trajectories 2, 7, and 8 occur prior to Aβ42 dimer dissociation into two monomers. At 500 ns of these trajectories, Aβ dimer (trajectories 1 and 5) or two dissociated monomers (trajectories 2, 7, and 8) are adhered to one or two adjacent sides of a lipid bilayer (Figure 7).

There is an additional trajectory-to-trajectory variability within this second pathway. Final configurations of Aβ42 dimers in trajectories 1 and 2 of system (v), for example, are unique as they reveal Aβ42 peptides in part stretched across the top of a bilayer, interacting with lipid head groups. These configurations somewhat resemble a postulated carpeting mechanism of lipid bilayer disruption, however, the main mode of Aβ42 dimer interaction with lipid bilayer in trajectory 2 is adhesion. Panel C of Figure S13 shows that trajectory 1 and even more so trajectory 2 are associated with the lowest nematic DMPC and CHOL order parameters. In trajectory 1, the low nematic order parameters may be mostly a consequence of Aβ42 dimer insertion into the lipid bilayer, which persists until 470 ns and disrupts lipid ordering within 400–500 ns (Figure 8C). In trajectory 2, however, with the lowest nematic DMPC and CHOL order parameters, the lipid ordering disruption cannot be due to Aβ42 dimer insertion, which is absent from time interval 400–500 ns. Trajectory 1 disrupts the DMPC density profile significantly more than trajectory 2 (Figure 5, panel E, blue versus orange lines) whereas the two trajectories disrupt the CHOL density profile to a similar extent (Figure 5, panel F, blue versus orange lines). Thus, Aβ42 dimer insertion distorts both the nematic lipid order parameters and the density profile of a bilayer.

We then reasoned that carpeting events may be quantified by monitoring time evolution of the number of Aβ42 dimer residues in contact with CHOL headgroups (O atoms) of those CHOL molecules whose directors deviate from the normal to the bilayer in both directions less than 45°, which are the CHOL molecules that contribute to bilayer features. Figure S16 shows per-trajectory time evolution of this quantity for lipids assembling in the presence of Aβ42 dimers. Trajectories 2 and 3 show enhanced numbers of thus selected CHOL headgroups at time intervals 280–360 ns and 220–380 ns, respectively. In trajectory 2, the time interval 280–360 ns overlaps with an insertion event at 230–320 ns, indicating that partial carpeting and insertion events can coexist, which makes it challenging to distinguish the effects of insertion from the effects of carpeting on bilayer properties. In trajectory 3, the partial carpeting event at 220–380 ns coexists with adhesion and is not associated with a particularly low nematic order parameter (Figure S13).

The average instantaneous number of DMPC and CHOL lipids in contact with Aβ42 monomers and dimers in Figure S17 indicates that each peptide in systems (iii) and (v) is in contact with 3–7 and 0–2 different DMPC and CHOL molecules, respectively. However, trajectory-to-trajectory variability of these numbers is significant. Notably, of all trajectories of system (v), trajectories 1–3 are associated with the highest number of CHOL and DMPC molecules per peptide in contact with Aβ42 dimers. A high number of CHOL contacts is expected for Aβ42 dimer inserted into CHOL-rich domain of a bilayer (trajectory 1) whereas a high number of DMPC contacts is consistent with Aβ42 dimer partially spread over the top of a bilayer as a carpet (trajectories 2–3). Movies of all trajectories corresponding to systems (i), (iii), and (v) are available as Supporting Information material.

## 3 Discussion and Conclusions

In this work, Aβ42 monomer and dimer association with a mixture of 100 initially free lipids (68 DMPC, 30 CHOL, and 2 GM1) is examined by explicit-solvent all-atom MD simulations to discern the effect of lipids on Aβ42 monomers and dimers, and vice versa. For each of the five different systems under study: (i) 100 lipids, (ii) Aβ42 monomers, (iii) Aβ42 monomers with 100 lipids, (iv) Aβ42 dimers, and (v) Aβ42 dimers with 100 lipids, we acquire and analyze 10 replica MD trajectories, to reveal that lipids assemble into a lipid bilayer-like structures with a central CHOL-rich domain that resembles a lipid raft. The bilayer-like assemblies observed in our simulations are completely immersed in the solvent and are characterized by round micelle-like edges with lipid head groups exposed to the solvent to minimize the solvent exposure of hydrophobic lipid tail groups. While it is unclear whether the CHOL domain in lipid bilayer-like structures forms due to a limited size and number of lipids in our simulations, our approach allows for Aβ42 monomer and dimer association with lipids to be investigated free of assumptions or simulation constraints.

We characterize lipid bilayer-like structures in the absence and presence of Aβ42 using configurations within 400–500 ns of all MD trajectories that converged into a single Aβ42-lipid cluster. Only 2 out of 10 and 1 out of 10 MD trajectories of systems (i) and (iii), respectively, are excluded from the analysis. Extending per-trajectory simulation time might result in full convergence of all trajectories, potentially leading to sharper density profiles and increased order parameters of the lipid bilayer without affecting the key findings of this study. Regardless of the above limitations, our MD approach of using free lipids to form a lipid bilayer-like assembly featuring a lipid raft-like domain can be used to investigate other amyloidogenic proteins and their association with lipids, which underlies various amyloidoses.

Our results show that lipids exert opposite effects on Aβ42 monomers and dimers. Lipids increase β-strand content and tertiary contacts in Aβ42 monomers while weakening both the tertiary and quaternary contacts in Aβ42 dimers, resulting in their partial dissociation. CHOL emerges as an important component of lipids that interacts more avidly with Aβ42 dimers than with monomers. Aβ42 monomer, which remains adjacent to a lipid micelle or a bilayer in all 10 replica trajectories, thus exhibits a single pathway of association with lipids. This adhesion of Aβ42 monomers to the edges of a bilayer shields hydrophobic lipid tails from the solvent, leading to an increase in the population of lipids that contributes to a bilayer-like characteristics, resulting in an increased order within a lipid bilayer. Lipids, in turn, render Aβ42 monomer less disordered and, by implication, more inert, hindering its interactions with a bilayer.

In contrast to Aβ42 monomer, Aβ42 dimer exhibits multiple pathways of association with lipids. While also sharing the same pathway as monomers as the dominant mode of coassembly, our simulations reveal probabilistic events of an intermittent spontaneous insertion of Aβ42 dimer into a lipid bilayer, persisting for at least 100 ns, in 5 out of 10 replica trajectories. An insertion event is here defined by Aβ42 dimer embedded into both leaflets of the bilayer-like assembly simultaneously. In addition, Aβ42 dimer exerts a carpeting effect by partially spreading itself across the top of a lipid bilayer, interacting mostly with lipid heads. This effect is observed in only 2 of 10 replica trajectories and overlaps with an insertion event or adhesion. Insertion and/or carpeting events cause a mechanical perturbation of a lipid bilayer, resulting in a destabilization which may directly increase membrane permeability to calcium influx or increase the membrane tension in a membrane that activates various mechanosensitive receptors in a membrane.^24^ These contrasting effect of monomers versus dimers on a lipid bilayer are consistent with experimental study by Maiti and collaborators who reported that thermodynamically stable Aβ42 monomers possess a much lower membrane affinity than a mixture of monomers and small oligomers, including dimers.^27^

Our MD study supports the hypothesis that Aβ42 dimers interact with free lipids distinctly from Aβ42 monomers, providing a plausible mechanistic explanation why dimers but not monomers are capable of damaging lipid bilayers. Lipids are shown to partially destabilize dimers, although the majority of dimers remains stable on the time scale of 500 ns. In turn, Aβ42 dimers perturb the integrity of a lipid bilayer via an insertion into a CHOL-rich domain of a bilayer-like structure, which is here observed to be a stochastic event occurring during Aβ42 dimer association with free lipids. Because dimer insertion events may be hindered by a partial lipid-induced dimer destabilization, dimer accumulation may be needed for the insertion events to occur. Our findings also imply that CHOL-rich lipid raft is instrumental for Aβ42 dimer insertion. Aβ42 oligomer-mediated damage to lipid vesicles, most likely due to oligomer penetration of the bilayer, has been observed *in vitro*.^58,59^ Our work here demonstrates that Aβ42 dimers coassembling with free lipids can get spontaneously inserted into lipid-bilayer-like structures, which is a prerequisite for Aβ42 oligomer-induced disruption in various environments, including synaptic clefts where increased concentrations of Aβ oligomers trigger a cascade of events leading to AD.

## 4 Methods

### 4.1 Molecular Dynamics Simulations

Fully atomistic MD simulations in explicit solvent are performed with GROMACS 2021.1 ^64–70^ using CHARMM36m force field with its specific TIP3P water model for simulations of Aβ42 monomers and dimers.^71–74^ CHARMM36 force field for lipids is used to model dimyristoylphosphatidylcholine (DMPC), CHOL and GM1.^75–77^ In a recent study assessing various lipid force fields for MD simulations, CHARMM36 has been shown to yield the best description of DMPC/CHOL systems of lipids.^78^ Four sets of MD trajectories of Aβ42 monomer and Aβ42 dimer in pure water and water with 100 lipid molecules (68 DMPC, 30 CHOL, and 2 GM1) in 150 mM NaCl are acquired. In addition, 10 MD trajectories of 100 lipids alone are acquired as a control system. Ten distinct MD trajectories, each 500 ns long, of the same system are collected to enhance the sampling and feed the statistical analysis, resulting in 20 μs of MD in total. Ten Aβ42 monomer or dimer conformations are derived from trajectories generated by discrete molecular dynamics (DMD) simulations with DMD4B-HYDRA force field,^79–81^ followed by a conversion to an all-heavy-atom conformations using in-house software *protsView*, as reported previously.^33,36^ Monomer and dimer conformations obtained by DMD4B-HYDRA simulations and converted to all-heavy-atom representation are selected at random with a sole purpose to create 10 initial conformations for 10 replica MD trajectories per system. In all production runs, Aβ42 monomers and dimers are modeled using CHARMM36m. Each selected conformer is placed into the center of a (12.5 nm)^3^ simulation box to ensure that the water layer in all six directions is at least 2–3 nm to avoid peptides interacting with their periodic images. The same simulation box size is chosen for all systems in our study, which ensures the same lipid molar concentration in systems (i), (iii), and (v). The N- and C-termini of each Aβ42 molecule correspond to ${\mathrm{NH}}_{3}^{+}$ and COO^–^, respectively. Three Na^+^ ions are added for each Aβ42 peptide to neutralize the system. The addition of hydrogen atoms to the Aβ42 monomer or dimer conformation, the addition of ions to the solvent, and the resolution of atom collisions is performed within GROMACS during the preparation and energy minimization steps. The Verlet cutoff scheme^82^ and a time step of 2 fs are used during the equilibration and production steps. Energy minimization is performed with the steepest descent method for 100000 steps and is followed by a 200 ps–long equilibration step at 310 K and 1.0 bar. All production runs use the Nosé-Hoover thermostat^83,84^ and the Parrinello–Rahman barostat.^85^

### 4.2 Root Mean Square Deviation and Fluctuation

The Root Mean Square Deviation (RMSD) serves as a metric to evaluate the convergence of our MD simulations. The calculation of RMSD is initiated by aligning the conformation at time *t* with a reference structure at *t* = 0 using the least-squares fitting. Subsequently, the RMSD is defined as

1 $$\mathrm{RMSD}\left(t\right)=\sqrt{\frac{1}{M}\overset{N}{\underset{i=1}{\sum }}m_{i}{\left|{\mathrm{r⃗}}_{i}\left(t\right)-{\mathrm{r⃗}}_{i}\left(0\right)\right|}^{2}}$$

Here, *M* represents the total mass of the system, defined as $M=\overset{N}{\underset{i=1}{\sum }}m_{i}$ , and ${\mathrm{r⃗}}_{i}\left(t\right)$ is the position of atom *i* at time *t*. For our analysis, the backbone of the protein was used for the RMSD calculation. The calculations are done over for all ten 500 ns-long trajectories of each relevant set of simulations: Aβ42 monomer and dimer in the absence and presence of lipids.

Residue-wise flexibility of Aβ42 is quantified using C_α_ root-mean-square fluctuations (RMSF) calculated using GROMACS module ’rmsf’. Analyses are performed over the 400–500 ns interval with frames sampled every 1 ns, and RMSF values computed on a per-residue basis. For Aβ42 dimers, RMSF is calculated separately for each chains and subsequently averaged residue-wise. Final RMSF values are obtained by averaging across all the trajectories and frames, with variability reported as the standard error of mean.

### 4.3 N-Terminal to C-Terminal Distance

The distance between the N-terminal and C-terminal (N–C distance) of the peptide provides an alternative metric for evaluating the convergence of our simulations. To determine the N–C distance, we calculated the distance between the C_α_-atom of the first residue and the C_α_-atom of the last residue in each chain of Aβ42 using the Gromacs tool ’pairdist’. This measurement was performed for all 10 500 ns long independent MD trajectories for both Aβ42 monomer and dimer in the absence and presence of lipid molecules.

### 4.4 Minimal Distance between Two Peptides

To monitor a potential dissociation of Aβ42 dimer into two monomers along MD trajectories, we track a minimal interpeptide distance between the two peptides in a dimer, which is calculated as a minimum over all interpeptide distances between pairs of non-hydrogen atoms using the GROMACS tool ’mindist’. Two peptides are considered to form a dimer if their minimal interpeptide distance is ≤0.5⁡nm .

### 4.5 Aβ42 Secondary Structure

The secondary structure of Aβ42 monomers and dimers in the absence and presence of lipids was analyzed using the STRIDE algorithm^86^ implemented in Visual Molecular Dynamics (VMD) software package.^61^ For each of the four systems under study (monomers and dimers, each in the absence and presence of lipids), two types of secondary structure were derived: (i) the average coil, strand, turn, and helical content per peptide, and (ii) the average per-residue coil, strand, turn, and helical propensity. Notably, the helical secondary structure in our calculation represents a sum of the three types of helices: α-helix (the dominant contribution), π-helix, and 3–10 helix. The secondary structure analysis was performed on all monomer or dimer conformations within the time interval 400–500 ns of each of the ten MD trajectories per system using time frames that were 1 ns apart. For each system, the final values were obtained by averaging over time frames of each trajectory, followed by the average over ten independent trajectories. The error bars represent the standard error of the mean (SEM) values.

### 4.6 Aβ42 Tertiary and Quaternary Structure

Using VMD, contact maps were derived to characterize the tertiary and quaternary contacts in Aβ42 monomers and dimers. A pair of amino acids is considered to be in contact if the distance between the respective C_α_ atoms is less than 6 Å. If the two amino acids belong to the same peptide, the contact is recorded as a tertiary contact; otherwise, it is recorded as a quaternary contact. The C_α_-C_α_ cutoff of 6 Å criterion is used to construct a two-dimensional matrix (map) that captures the probability of a contact between two residues. Tertiary (intramolecular) contact maps for monomers and tertiary and quaternary (intermolecular) contact maps for dimers were calculated for both conditions, the absence and presence of lipids, using time frames separated by 1 ns within 400–500 ns of each of the ten MD trajectories per system. For each system, contact maps were computed for the final 100 ns of each trajectory and then averaged across ten independent trajectories. Each element (i,j) of the contact map represents the probability of contact between the amino acids i and j, along with the standard error of mean (SEM), plotted above and below the main diagonal, respectively. To assess the effect of lipids on the tertiary and quaternary structure, we also computed a difference contact map by subtracting the contact map obtained in the absence of lipids from the contact map obtained in the presence of lipids.

### 4.7 Per-Residue Aβ42 Contacts with Lipids

To gain insight into residue-specific interactions between Aβ42 and lipid molecules, we calculated the average per-residue contact frequencies between Aβ42 and DMPC or CHOL. Atomic contacts are defined using a distance threshold of 3.5 Å between any non-hydrogen atom of a specific amino acid residue in Aβ42 and any non-hydrogen atom of DMPC or CHOL. The average number of atomic contacts between a specific residue of Aβ42 and DMPC or CHOL was calculated using time frames within 400–500 ns, separated by 1 ns, of all ten MD trajectories per system. In the case of Aβ42 dimers, the average number of atomic contacts represents an average over the two peptides. The resulting per-residue contact frequencies are obtained by normalizing the average numbers of contacts by the number of lipid molecules in each system, i.e. 68 for DMPC and 30 for CHOL, to ensure comparability between DMPC and CHOL.

### 4.8 Per-Residue Residence Times

For 400–500 ns of each all MD trajectories corresponding to systems (iii) and (v), residence times of lipid-Aβ42 contacts are computed on a per-residue basis using a heavy-atom distance cutoff of 0.35 nm. By definition, a contact between a residue of interest and a lipid molecule occurs when any heavy atom of the residue is within the cutoff distance of any heavy atom of the lipid. For each residue-lipid pair, a binary contact time series, i.e. a string of values 0 and 1 (indicating the absence and presence of at least one contact, respectively), is constructed across the frames, and residence times are defined as continuous time intervals during which a lipid molecule remains in an uninterrupted contact with the residue. Each continuous contact segment is treated as an independent residence event, and its duration is determined from the simulation time resolution of 50 frames per nanosecond, i.e. *δt* = 20 ps. For each residue, residence times are averaged over all contact events and lipid molecules. This procedure is performed independently for each trajectory, and final residue-wise mean residence times are obtained by averaging over the ensemble of trajectories. The corresponding SEM value reflect both time and ensemble statistics. For dimers, residue-wise residence times are averaged over the two peptides in a dimers.

### 4.9 Cluster Size Distributions

The clustering behavior of three systems: lipids alone, Aβ42 monomers with lipids, and Aβ42 dimers with lipids is examined using a 3.5 Å threshold (cutoff) within GROMACS tool ’clustsize’, whereby a molecule (Aβ42, DMPC, CHOL, or GM1) is identified as a part of the same cluster if any of its non-hydrogen atoms are within a distance of 3.5 Å from any of the non-hydrogen atoms of another molecule. The cluster size distributions are calculated using time frames, separated by 1 ns, within 400–500 ns of each trajectory, followed by an ensemble average over ten trajectories per system. The size distributions of DMPC- and CHOL-only clusters are calculated in the same way, as described above.

### 4.10 Density Profile of a Lipid Bilayer

The formation of a lipid bilayer is examined for three systems: (i) lipids alone, (iii) Aβ 42 monomers with lipids and (v) Aβ42 dimers with lipids, whereby all time frames, separated by 1 ns, within 400–500 ns of replica MD trajectories per system are used in the analysis. For each time frame, a normal to a lipid bilayer, hereby referred to as a director, is calculated using function ’compute_directors’ within ’compute_nematic_order’ from python software package MDTraj.^87^ This calculation is explained in detail in section 4.11. Then, atomic positions of the selected headgroup atoms, i.e. N atoms of DMPC and O atoms of CHOL, are projected onto the bilayer director to obtain a one-dimensional distance distribution, corresponding to the headgroup density profile of the bilayer. We first calculate per-trajectory headgroup density profiles, followed by an ensemble average over all replica MD trajectories. Selected trajectories, trajectories 3 and 4 in system (i) and trajectory 5 of system (iii), are excluded from the ensemble average if the molecules (Aβ42, DMPC, CHOL, GM1) formed two or more clusters within time interval 400–500 ns.

### 4.11 Nematic Lipid Order Parameters

We calculate the nematic order parameter, *S*_2_, using function ’compute_nematic_order’ within MDTraj^87^ to quantify the orientational order of lipids in a bilayer. For each DMPC or CHOL lipid molecule *i* in a lipid assembly of interest, the molecular orientation vector **u**_*i*_ is determined from a molecular inertia tensor defined as

2 $$I_{ab}=\overset{M}{\underset{k=1}{\sum }}m_{k}\left(r_{k}^{\prime 2}\delta_{ab}-r_{k,a}^{\prime }r_{k,b}^{\prime }\right)$$

where *m*_*k*_ and $r_{k}^{\prime }$ denote the mass and position of each non-hydrogen atom *k* relative to the center of mass of lipid *i*, respectively, and δ_*ab*_ is the Kronecker delta. The molecular inertia tensor is then diagonalized, resulting in three eigenvalues and eigenvectors corresponding to the principal moments of inertia and respective axes of rotation. The director of lipid *i*, unit vector **u**_*i*_, is identified as the eigenvector associated with the smallest eigenvalue of *I*_*ab*_, i.e. the smallest rotational moment of inertia around the long molecular axis of the molecule.

A nematic order parameter of the lipid assembly is derived from the alignment tensor, which is calculated using lipid directors **u**_*i*_ as

3 $$Q_{ab}=\frac{1}{2N}\overset{N}{\underset{i=1}{\sum }}\left(3u_{i,a}u_{i,b}-\delta_{ab}\right)$$

where *N* is the number of lipids in the assembly. The nematic order parameter *S*_2_ is identified as the largest eigenvalue of *Q*, and the corresponding eigenvector is used as a self-consistent global nematic director of the lipid assembly. This formulation is mathematically equivalent to the ensemble average of the second Legendre polynomial, *S*_2_ = ⟨(3 cos^2^θ – 1)/2⟩, where θ is the angle between each lipid director and the global nematic director. Because a spontaneously formed lipid assembly may exhibit tilt and/or curvature, the order parameter is computed globally over all lipids without separating leaflets.

Average DMPC and CHOL nematic order parameters are calculated using only DMPC or only CHOL lipid molecules within the assembly, resulting in DMPC and CHOL order parameters, respectively. In addition, the above calculation of tensor *Q* is performed using all DMPC and CHOL lipids in the assembly. The diagonalization of this tensor results in the eigenvector associated with the largest eigenvalue, i.e. a director, which is identified as the normal to the bilayer, unit vector **n**, for all configurations in which lipids and Aβ42 form a single cluster, and used in the calculation of the lipid density profiles and projections of lipid directors onto the bilayer normal (sections 4.10 and 4.12).

For each time frame within 400–500 ns of each MD trajectory of interest, the lipid order parameters are calculated and averaged over all time frames, separated by 1 ns. The final order parameter values are thus obtained as time and ensemble averages over the relevant trajectories per system and the SEM values reflect both time and trajectory-to-trajectory variability.

### 4.12 Projections of Lipid Directors on the Bilayer Normal

For systems (i), (iii), and (v), DMPC and CHOL lipid directors **u**_*i*_ and the normal to the lipid bilayer **n** are calculated as described in section 4.10. A normalized distribution of all projections of DMPC and CHOL lipid directors onto the bilayer normal, **u**_*i*_ ·**n**, is calculated. These projections are collected over all time frames within 400–500 ns of all trajectories, except for trajectories 3 and 4 of system (i) and trajectory 5 of system (iii), to generate a probability density distribution of lipid directors using the Gaussian kernel density estimation.

### 4.13 Carbon–Carbon and Deuterium Order Parameters

The orientational ordering of lipid acyl chains is quantified using both carbon–carbon (*S*_*cc*_) and deuterium (*S*_*cd*_) order parameters. For each trajectory frame, the global director is determined, as described in 4.11. In the case of *S*_*cc*_, bond vectors are defined between consecutive carbon atoms along each acyl chain (C_*i*_ to C_*i*+1_) and the angle between each bond vector and the global director is calculated. In the case of *S*_*cd*_, the angles between all C–H bond vectors and the global director are calculated. The two order parameters are then calculated as averages:

4 $$\left\langle S\left(i\right)\right\rangle =\left\langle \frac{1}{2}\left(3\cos^{2}\theta_{i}-1\right)\right\rangle$$

where θ_*i*_ is the angle between the bond vector associated with carbon C_*i*_ and the global director. The final averages and SEM values are obtained using all time frames between 400 and 500 ns of all relevant trajectories.

### 4.14 Hexatic Order Parameter

The lateral packing of lipid molecules within each leaflet is quantified using the hexatic order parameter, Ψ_6_, defined as a complex quantity whose phase reflects the local bond orientation and whose magnitude reflects the degree of 6-fold bond-orientational order:

5 $$\Psi_{6}\left(i\right)=\frac{1}{N_{i}}\overset{N_{i}}{\underset{j=1}{\sum }}e^{6i\theta_{ij}}$$

where θ_*ij*_ is the angle between the vector connecting lipid *i* to its neighbor *j* and the *x*-axis of the projected two-dimensional bilayer plane. The bilayer plane is defined by two orthonormal basis vectors constructed from the bilayer director, and θ_*ij*_ is measured after projection of lipid head coordinates onto this plane. The calculations are performed using python library *freud*.^88^ For each trajectory, lipid headgroup atoms (N for DMPC and O for CHOL) are selected to represent lipid positions. At each frame, the lipid bilayer is divided into top and bottom leaflets based on the position of lipid headgroups upon its projection onto the global director. Within each leaflet, lipid coordinates are projected onto the lipid bilayer plane to obtain two-dimensional positions. The hexatic order parameter is then computed individually for DMPC and CHOL. For each lipid, six nearest neighbors of the same lipid type are identified, and the local hexatic order parameter Ψ_6_(*i*) is first calculated as a complex quantity. For each frame, hexatic order parameter magnitudes, i.e., |Ψ_6_(*i*)| values, are then averaged over all lipids of given type within each leaflet. These per-frame leaflet averages are subsequently averaged over time frames within 400–500 ns of each trajectory to obtain per-trajectory estimates for DMPC and CHOL hexatic order parameters in the top and bottom leaflets. Final reported average values correspond to averages over the per-trajectory top- and bottom-leaflet values from all relevant MD trajectories.

### 4.15 Solvent Accessible Surface Area

The time evolution of Solvent accessible surface area (SASA) is calculated for the Aβ42-lipid systems to quantify the solvent exposure of peptide and lipid components using GROMACS. Total Aβ42 peptide SASA was calculated by considering all heavy atoms. Hydrophobic SASA is computed by restricting the calculation to the hydrophobic residues of Aβ42, thereby quantifying the solvent exposure of hydrophobic regions of the peptide. To characterize lipid-specific contributions, SASA is also evaluated separately for lipid headgroup and tailgroup regions. Headgroup SASA is defined using the P atom and N atom for DMPC and O atom for CHOL. Tailgroup SASA is defined using the acyl chain carbon atoms of DMPC and all nonheadgroup atoms of CHOL.

### 4.16 van der Waals and Electrostatic Potential Energies

van der Waals and electrostatic interaction energies between Aβ42 and lipids are calculated within GROMACS. Trajectories are sampled at 1 ns intervals, and interaction energies are evaluated for each frame using the same force field parameters and simulation settings as in the production simulations. Energy decomposition is performed by defining separate groups for the peptide, DMPC, and CHOL, enabling extraction of pairwise interaction terms. Short-range van der Waals interaction energies are obtained from the Lennard-Jones potential (LJ-SR), while the electrostatic interaction energies are computed from the short-range Coulomb potential (Coul-SR), consistent with the Verlet cutoff scheme used in the production simulations. For Aβ42 dimers, the energies are averaged over the two peptides.

### 4.17 Number of Protein Residues in Contact with Lipid Headgroups/Tailgroups

Residue-lipid contact analysis is performed using MDTraj. All Aβ42 atoms other than hydrogens are included in these calculations. A contact is defined based on a distance cutoff of 0.35 nm between any protein atom and selected lipid atoms. The N atom of DMPC and the O atom of CHOL are considered as the head groups, whereas all carbon atoms of DMPC acyl chains and all CHOL atoms other than the O-group are considered as tailgroups. For each trajectory frame, contacts are recorded at the residue level: a residue is in contact with the lipid headgroup or tailgroup if at least one of its heavy atoms satisfied the above distance criterion. The total number of Aβ42 residues in contact with lipid headgroups or tailgroups is then computed for each peptide and averaged over the two peptides in a dimer.

## Supplementary Material









## Data Availability

The data produced in this study are available on request from the corresponding author.

## Notes

Movies of 30 MD trajectories corresponding to system (i) (ZIP)
Movies of 30 MD trajectories corresponding to system (iii) (ZIP)
Movies of 30 MD trajectories corresponding to system (v) (ZIP)
Supporting results: convergence of MD trajectories; the effect of lipids on the secondary, tertiary, and quaternary structure of Aβ42 conformers, supporting figures: time evolution of RMSD values; N–C distances, and RMSF values; per-trajectory snapshots of Aβ42 monomer and dimer conformations at 500 ns; per-residue secondary structure propensities of Aβ42 conformers in the absence and presence of lipids; tertiary and quaternary contact maps; per-residue residence times; various cluster size distributions; time evolution of nematic lipid order parameters; per-trajectory nematic lipid order parameters; time evolution of 11 quantities for selected Aβ42 dimer-lipid trajectories; time evolution of key quantities involved in dimer insertion events and carpeting; per-trajectory numbers of lipids in contact with Aβ42 monomer and dimer (PDF)
