# Supplementary material for: Spontaneous Insertion of Aβ42 Dimers but Not Monomers into a Cholesterol-Rich Lipid Bilayer
Source: ACS Chem Neurosci. 2026 May 22;17(13):2469–85. doi: 10.1021/acschemneuro.5c00942 (PMC13329904; doi:10.1021/acschemneuro.5c00942)
Supplement: Supplementary file 4 [file cn5c00942_si_004.pdf]

Supporting Information: Spontaneous insertion of Aβ42 dimers but not monomers into a cholesterol-rich lipid bilayer

Rachit Pandey, Thomas Ruggiero, Brian Andrews, Brigita Urbanc*

Physics Department, Drexel University, 3141 Chestnut St, Philadelphia, 19104, PA, USA.

Supporting Results

Convergence of MD trajectories: RMSD, N-to-C distance, and RMSF values

The RMSD values for all four systems increase the most within the first 50 ns and appear to approach a steady state within the initial 200 ns, without showing any significant system-specific features (Fig. S1). An exception is the RMSD values of Aβ42 monomers, which exhibit somewhat smaller deviations from the average in the absence of lipids (panels A and B in Fig. S1). The N-C distance in Fig. S2 (A-D) exhibits even larger trajectory-to-trajectory variability, highlighting the importance of exploring multiple MD trajectories when studying IDPs. Importantly, N-C distance values take longer to equilibrate, in particular in the presence of lipids (for example, panels C and D in Fig. S2). Panels (E-F) in Fig. S2 show the N-C distance distributions of Aβ42 monomers in the absence and presence of lipids (panel E) as well as Aβ42 dimers in the absence and presence of lipids (panel F) using time frames within 400-500 ns of each trajectory. Whereas lipids do not significantly affect the average N-C distance in Aβ42 monomers (panel E), they increase the average N-C distance in Aβ42 dimers (panel F). While the average N-C distance is not affected by lipids for Aβ42 monomers, the distribution itself looks very different in the presence of lipids (panel E). Time evolution of RMSF values in Fig. S3 also demonstrates that lipids inhibit fluctuations in monomers more strongly than in dimers.

*Corresponding author: bu25@drexel.edu

The effect of lipids on Aβ42 per-residue secondary structure propensities

Lipids may affect the secondary structure of Aβ42 monomers and dimers in amino acid residue-specific way. We thus calculated per-residue secondary structure propensities for both Aβ42 monomers and dimers in the presence and absence of lipids. Per-residue secondary structure propensities of monomers and dimers are compared in Fig. S5, where the panels on the left correspond to lipids-free systems and the panels on the right correspond to systems with lipids. In the absence of lipids, per-residue coil propensities in Aβ42 dimers are lower than in Aβ42 monomers along the entire sequence with a few exceptions (Fig. S5). The main gain-of-structure in dimers relative to monomers can be seen in per-residue β-strand propensities, which are increased along the entire sequence except in region A30-I32, where monomers exhibit higher β-strand propensities than dimers. While dimers also exhibit slightly higher per-residue turn propensities than monomers (with an exception of region G33-G37), differences between monomers and dimers are smaller. In the presence of lipids, Aβ42 dimers exhibit higher per-residue coil propensities and lower per-residue β-strand and turn propensities than monomers (Fig. S5, right graph). The exceptions are only two regions, I32-M35 (within the MHR) and E11-H14, where relative to monomers, Aβ42 dimers exhibit higher β-strand and turn propensities, respectively. Relative to monomers, per-residue helical propensities in Aβ42 dimers, however, increase in the presence of lipids, which is particularly obvious within a broader central region (V12-N27) where monomers display almost no helical structure.

Fig. S6 demonstrates that lipids affect the per-residue propensities of Aβ42 monomers significantly more than per-residue propensities of Aβ42 dimers. In Aβ42 monomers, lipids decrease per-residue coil propensities and increase per-residue turn and/or β-strand propensities in regions A1-Y10, F19-E22, and N27-A30. In the presence of lipids, β-strand propensities in Aβ42 monomers increase within the broader N-terminal region (A1-G29) and assume the highest values within the CHC (F19-A21). Lipids tend to decrease per-residue β-strand propensities within the MHR and CTR (specifically, regions A30-I32 and V39-V40), resulting in a redistribution of β-strand structure from the C-terminus to the N-terminus. In the presence of lipids, per-residue turn propensities in N-terminal region A1-G9 of Aβ42 monomers increase. Although helical content is generally low, free lipids lead to a subtle decrease in per-residue helical propensities in region H14-F20 of Aβ42 monomers. Taken together, lipids reduce coil and increase turn and β-strand propensities, mostly within the N-terminal and central regions of Aβ42 monomers.

Fig. S6 also shows that per-residue secondary structure propensities in Aβ42 dimers are not strongly affected by the presence of lipids (right panels). In Aβ42 dimers, free lipids slightly increased per-residue coil propensities and decreased per-residue β-strand propensities along most of the sequence. Specifically, β-strand propensities decrease in regions Y10-V12, L34-V36, and V39-I41 when lipids are present. Per-residue turn propensities in Aβ42 dimers increase within regions H6-G9, E11-V12, I31-V36 and slightly decrease within the CFR in the presence of lipids. In dimers, lipids induce an increase in per-residue helical propensity within region H14-G25 and a notable decrease in region K28-V36. These data indicate that lipids mostly redistribute

the secondary structure propensities in Aβ42 dimers without strongly affecting the average secondary structure content.

The effect of lipids on Aβ42 tertiary and quaternary structure

To investigate how lipids affect tertiary and quaternary structure of Aβ42 monomers and dimers, we calculated intramolecular and intermolecular contact maps for systems (ii)-(v) as described in *Methods* (Fig. S7, panel A). In the absence of lipids, the most prominent intramolecular contacts in Aβ42 monomers form between regions L17-E22 and A30-G33, stabilizing the CFR, which is flanked between the CHC and MHR (Fig. S7, panel A, top left). Additional long-range tertiary contacts are formed between regions G9-V12 and G38-V40 as well as between R5-D7 and K16-V18. The strongest tertiary contact in Aβ42 monomers in the absence of lipids is V18-G33, contributing to the stability of the CFR loop. In the presence of lipids, the number of tertiary contacts in Aβ42 monomers increases and new prominent long-range contacts appear between regions R5-E11 and G38-V40, whereby residues H6 and G9 form particularly strong tertiary contacts with V39. Tertiary contact map of monomers in the presence of lipids features more ordered patterns that resemble anti-parallel β-sheets upon comparison to the tertiary contact map of monomers in the absence of lipids. These results suggest that lipids promote β-strand-rich conformations in Aβ42 monomers that are less accessible in purely aqueous environments.

In contrast to the effect of lipids on the tertiary structure of monomers, lipids inhibit the tertiary structure of Aβ42 dimers as reflected by a smaller number of tertiary contacts in the intramolecular map in the presence of lipids (Fig. S7, panel A, center). When lipids are not present, the strongest tertiary contacts in Aβ42 dimers form between regions G9-V12 and K16-F19 (Fig. S7, panel A, top center). These tertiary contacts disappear in the presence of lipids. Interestingly, tertiary contacts between regions L17-V24 and G29-L34 in dimers become stronger albeit reduced in numbers, indicating a more structured CFR in the presence of lipids. Under no-lipids conditions, N-terminal region D1-R5 in dimers makes long-range tertiary contacts with the MHR and CTR. These contacts are significantly weaker when lipids are present and compensated by shorter-range tertiary contacts between the D1-R5 region and CHC. This lipids-induced loss of long-range tertiary contacts indicates partial unfolding of peptides comprising a dimer, consistent with a significant increase in the N-C terminal distance (Fig. S2, panels C, D, and F).

In the presence of lipids, the number of quarternary contacts in Aβ42 dimers is reduced, indicating a loss of quaternary structure (Fig. S7, panel A, right). This observation is consistent with a previously observed lipids-induced decrease in the number of intermolecular contacts in Aβ42 dimers (Fig. 2 C-D). In the absence of lipids, quaternary contacts ranked by their strength are: V18-G33, I31-V36, L34-V39, A30-V36, M35-V39, S8-G37, Y10-L34, whereas in presence of lipids, the strongest quaternary contacts, in the ranking order, are: V18-F20, V18-F19, F19-F19, G33-G33, L34-L34, M35-M35, V36-V36, and G37-G37. Thus, lipids abolish quaternary contacts between the CHC and MHR as well as quaternary contacts between the MHR and CTR. Further, long-range quaternary contacts that the A1-R5 region makes with the MHR and also with the CTR in dimers in the absence of lipids are completely inhibited

by lipids. In contrast, quaternary contacts between pairs of CHCs as well as in-register, parallel strand-like contacts between the two MHRs are enhanced in the presence of lipids.

Difference maps obtained by subtracting a contact map of a system with lipids from the contact map of a system without lipids in Fig. S7B summarize the observations above. The tertiary difference contacts in monomers are dominated by red, which reflects the gain-of-contacts in the presence of lipids (Fig. S7B, left). The maximum and minimum difference tertiary contacts in monomers correspond to H6-G38 (strongest in the presence of lipids) and V18-G33 (strongest in the absence of lipids), respectively. In contrast, both tertiary and quaternary contacts in dimers are dominated by green, demonstrating lipids-induced loss of contacts in dimers (Fig. S7B, center and right). The maximum and minimum difference tertiary contacts in dimers are V18-G33 (strongest in the presence of lipids) and Y10-K16 (strongest in the absence of lipids), respectively. The maximum and minimum difference quaternary contacts in dimers are V18-F20 (strongest in the presence of lipids) and V18-G33 (strongest in the absence of lipids), respectively.

Supporting Figures

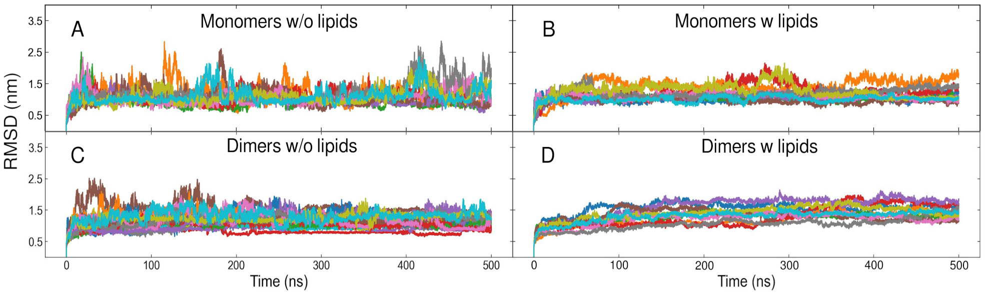

Fig. S1 Time evolution of the RMSD values of Aβ42 (A-B) monomers and (C-D) dimers in the (A,C) absence and (B,D) presence of lipids. Different trajectories are marked in distinct colors.

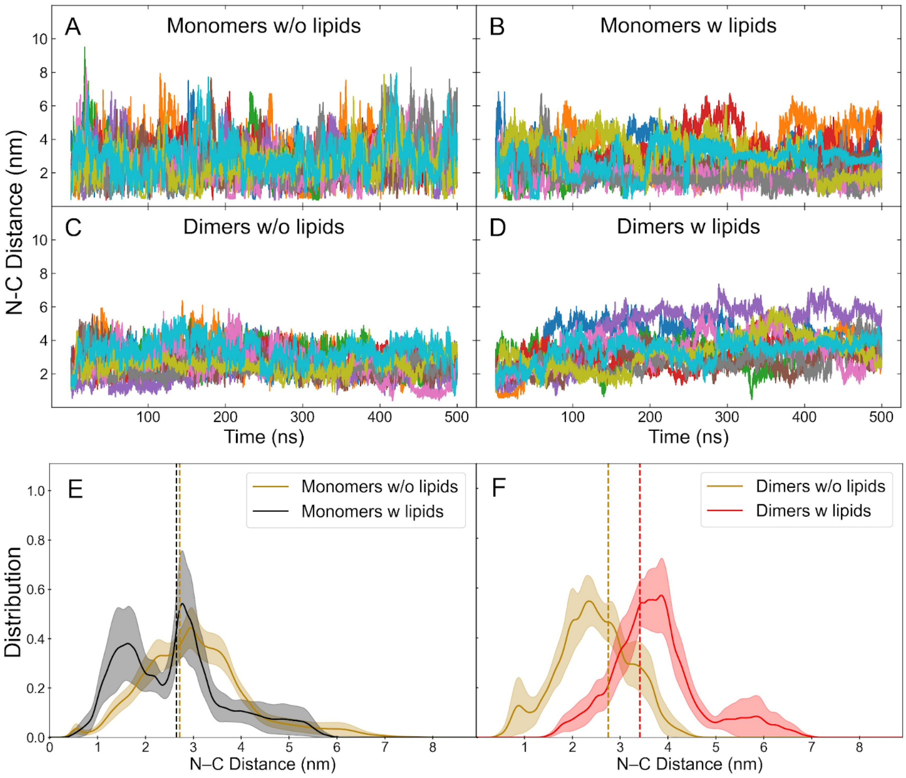

Fig. S2 (A-D) Time evolution of the N-terminal to C-terminal (N-C) distance for Aβ42 monomers in the (A) absence and (B) presence of lipids, and Aβ42 dimers in the (C) absence and (D) presence of lipids. Ten replica trajectories per system are displayed in distinct colors. (E-F) The effect of lipids on the probability distributions of the N-C distance for (E) monomers and (F) dimers (F). Probability distributions are based on N-C distance values within 400-500 ns of each of the ten respective replica trajectory per system. The error bars correspond to the SEM values obtained by the ensemble average over ten replica trajectories per system. The vertical dashed lines mark the average N-C distance values of (E) 2.72 ± 0.06 nm and 2.65 ± 0.20 nm for monomers in the absence and presence of lipids, respectively, and (F) 2.75 ± 0.11 nm and 3.41 ± 0.17 nm for dimers in the absence and presence of lipids, respectively. These error bars correspond to the SEM values obtained by statistics over 100 time frames per trajectory within 400-500 ns and the ensemble average over ten replica trajectories per system.

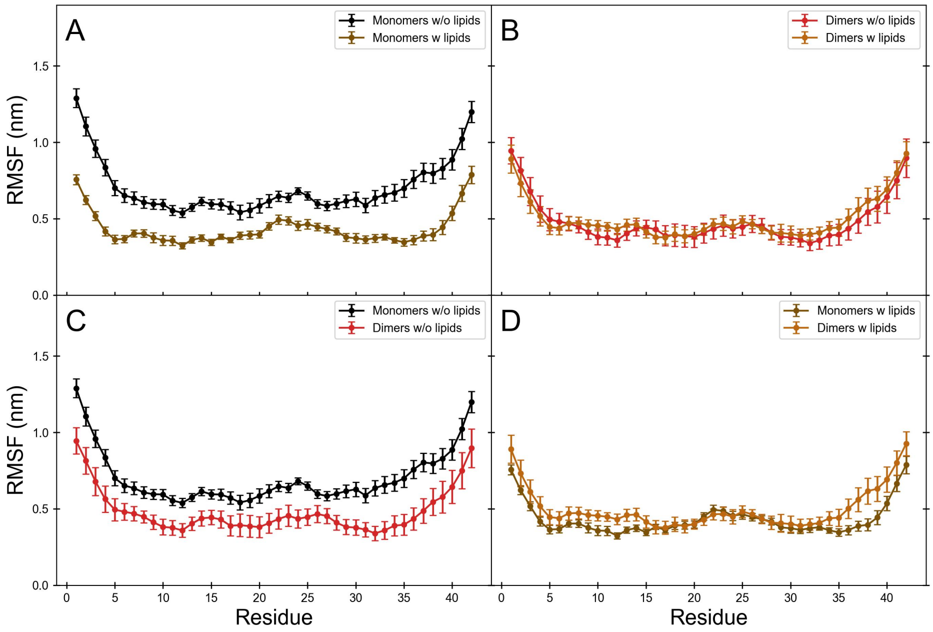

Fig. S3 Per-residue root mean square fluctuation (RMSF) values for Aβ42 (A) monomers with and without lipids and (B) dimers with and without lipids. Per-residue RMSF values for Aβ42 monomers and dimers in the (C) absence and (D) presence of lipids. For dimers, each RMSF value represents an average over the two peptide chains. The RMSF values are computed per trajectory by time-averaging over 400-500 ns, followed by the respective ensemble average over ten independent replica trajectories. Error bars correspond to SEM values, reflecting trajectory-to-trajectory variability.

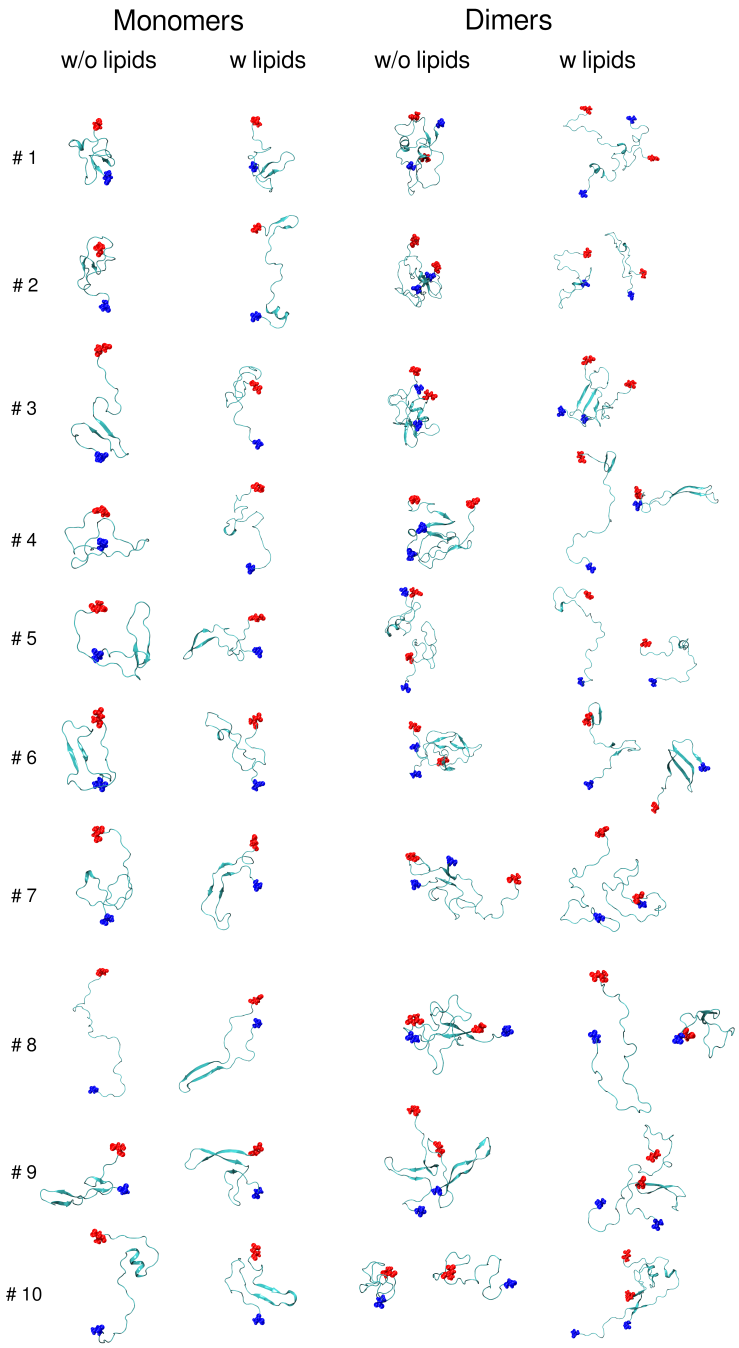

Fig. S4 Snapshots of Aβ42 monomers and dimers at 500 ns of each of the ten replica trajectories per system, both in the absence (w/o) and presence (w) of lipids. N- and C-termini of each Aβ42 are represented as red and blue beads, respectively.

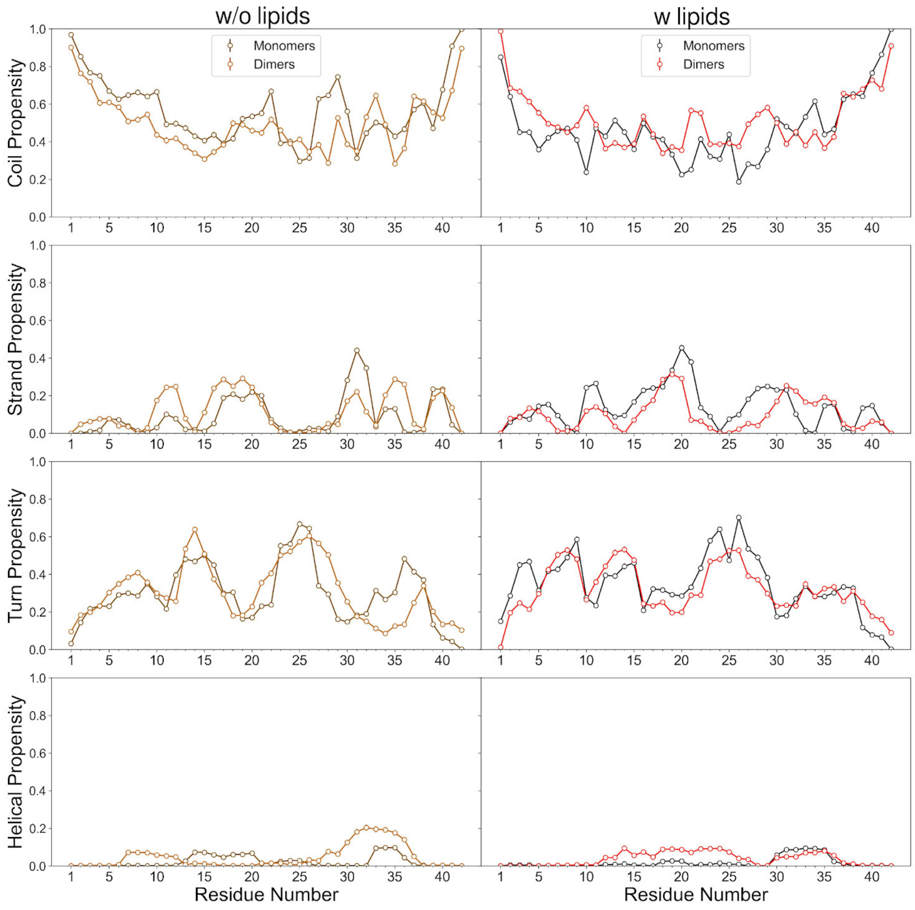

Fig. S5 Per-residue coil, strand, turn, and helical propensities in Aβ42 monomers and dimers in the absence (left panels) and presence (right panels) of lipids. The error bars correspond to SEM values.

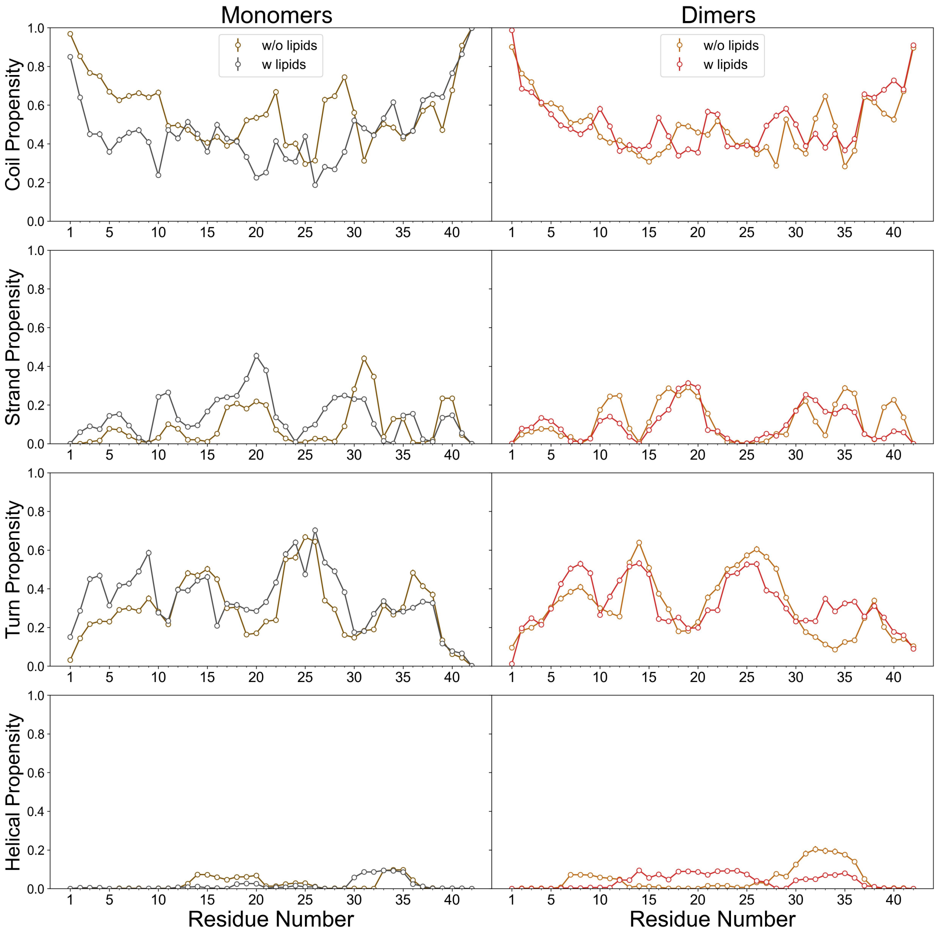

Fig. S6 Per-residue coil, strand, turn, and helical propensities in Aβ42 monomers (left panels) and dimers (right panels) in the absence and presence of lipids. The error bars correspond to SEM values.

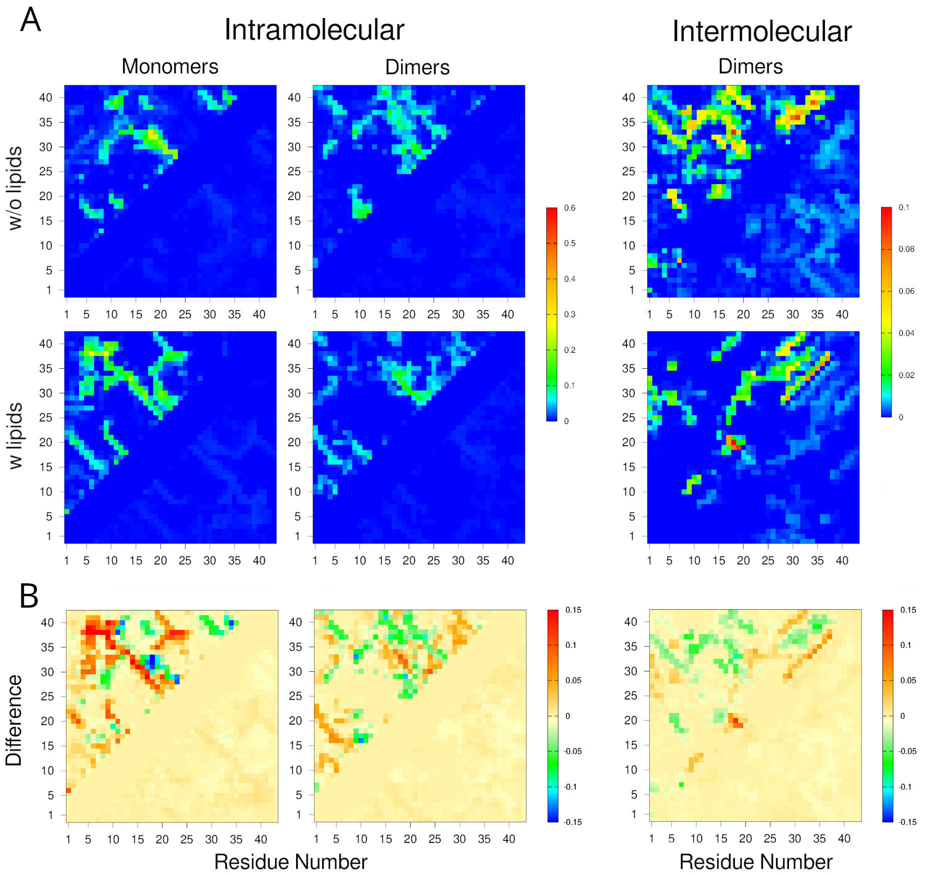

Fig. S7 The effect of lipids on tertiary and quaternary structure of Aβ42 monomers and dimers. Panels on the left and in the center show tertiary (intramolecular) contact probabilities in monomers and dimers, respectively. Quaternary (intermolecular) contact probabilities in dimers are displayed in the panels on the right. The top and middle rows correspond to conformations in the absence and presence of lipids, respectively. The bottom row shows lipids minus no-lipids differences of contact probabilities to elucidate the tertiary and quaternary structure changes due to lipids. The color scales show the contact probabilities.

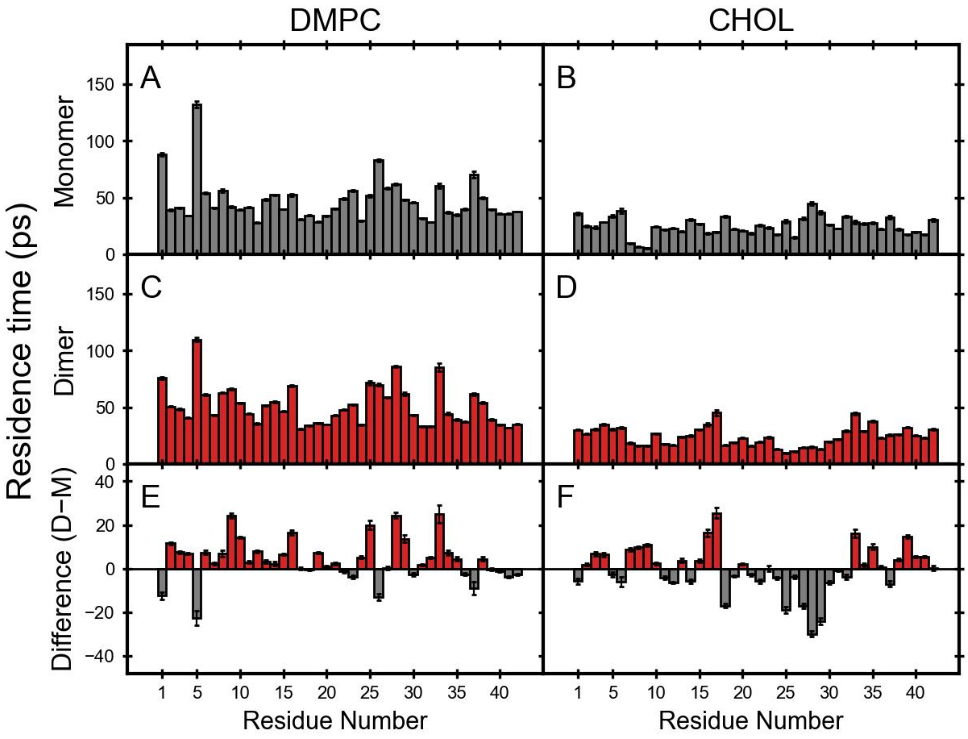

Fig. S8 Average residence times of contacts between Aβ42 monomer and dimer residues and DMPC or CHOL lipids.

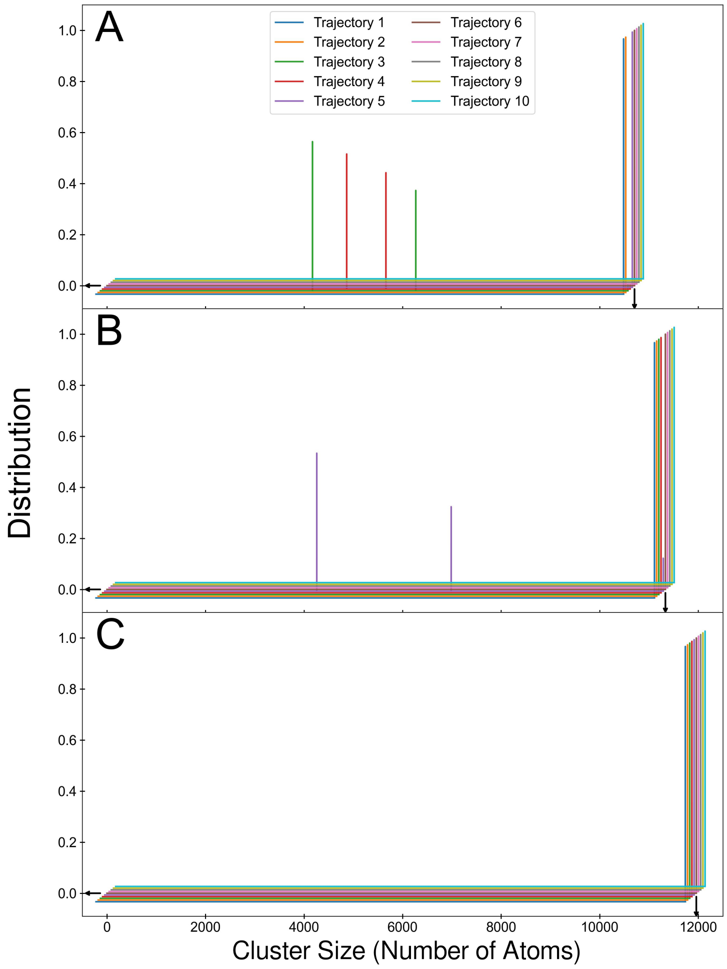

Fig. S9 The cluster size distribution of three systems: (A) lipids-only, (B) lipids with Aβ42 monomers, and (C) lipids with Aβ42 dimers. For each system, the maximal number of atoms corresponding to a single cluster is marked by an arrow on the x-axis: 10706, 11333, 11960 atoms for lipids-only, lipids with monomers, and lipids with dimers, respectively. The distributions of distinct trajectories are shifted along x- and y-axes to avoid overlap.

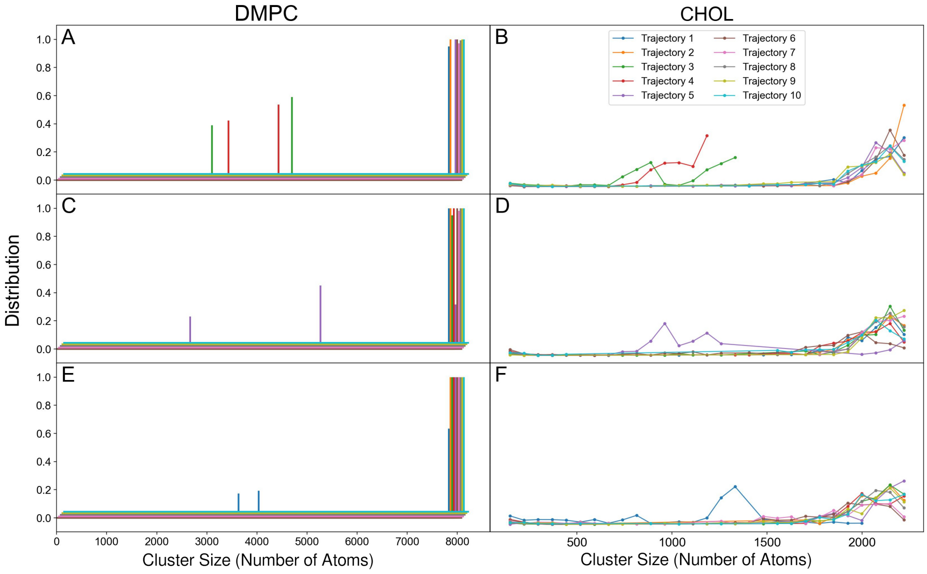

Fig. S10 The cluster size distribution of DMPC molecules (A,C,E) and CHOL molecules (B,D,F) for the three systems: (i) lipids-only, (iii) lipids with Aβ42 monomers, and (v) lipids with Aβ42 dimers. The distributions of distinct trajectories are shifted along x- and y-axes to avoid overlap.

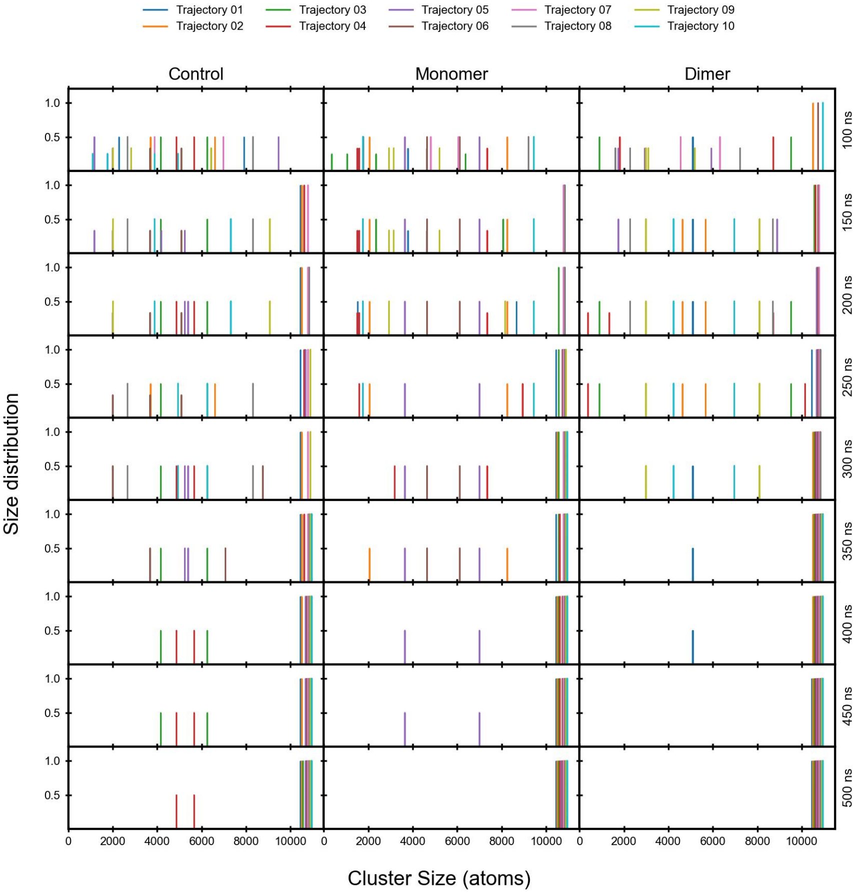

Fig. S11 Time evolution of lipid cluster size distributions for all lipids (DMPC, CHOL, and GM1) in system (i) without Aβ (left column), system (iii) with Aβ42 monomers (middle column), and system (v) with Aβ42 dimers (right column). Lipid cluster size distributions are calculated for each trajectory individually every 50 ns, starting at 100 ns.

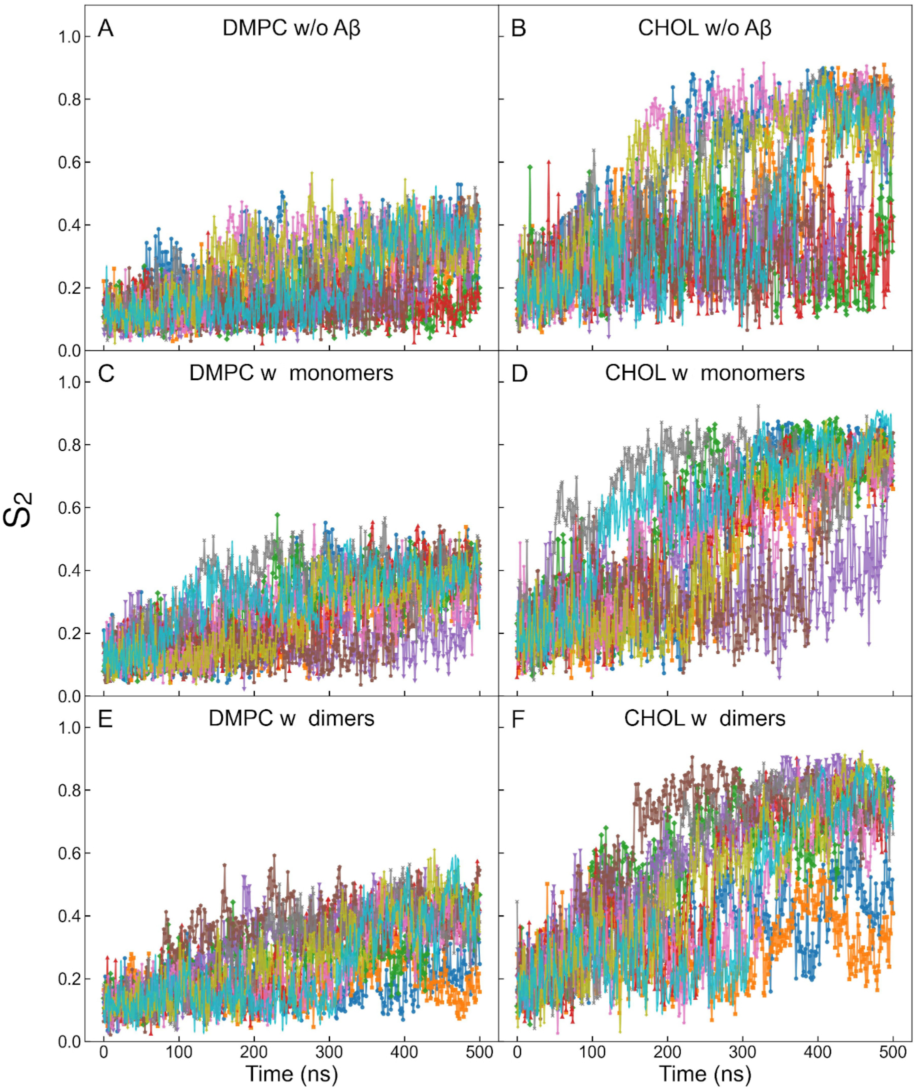

Fig. S12 Time evolution of the nematic order parameter of DMPC and CHOL molecules (A-B) in the absence of Aβ, (C-D) in the presence of Aβ42 monomers, and (E-F) in the presence of Aβ42 dimers. Ten replica trajectories for each system are represented with distinct colors.

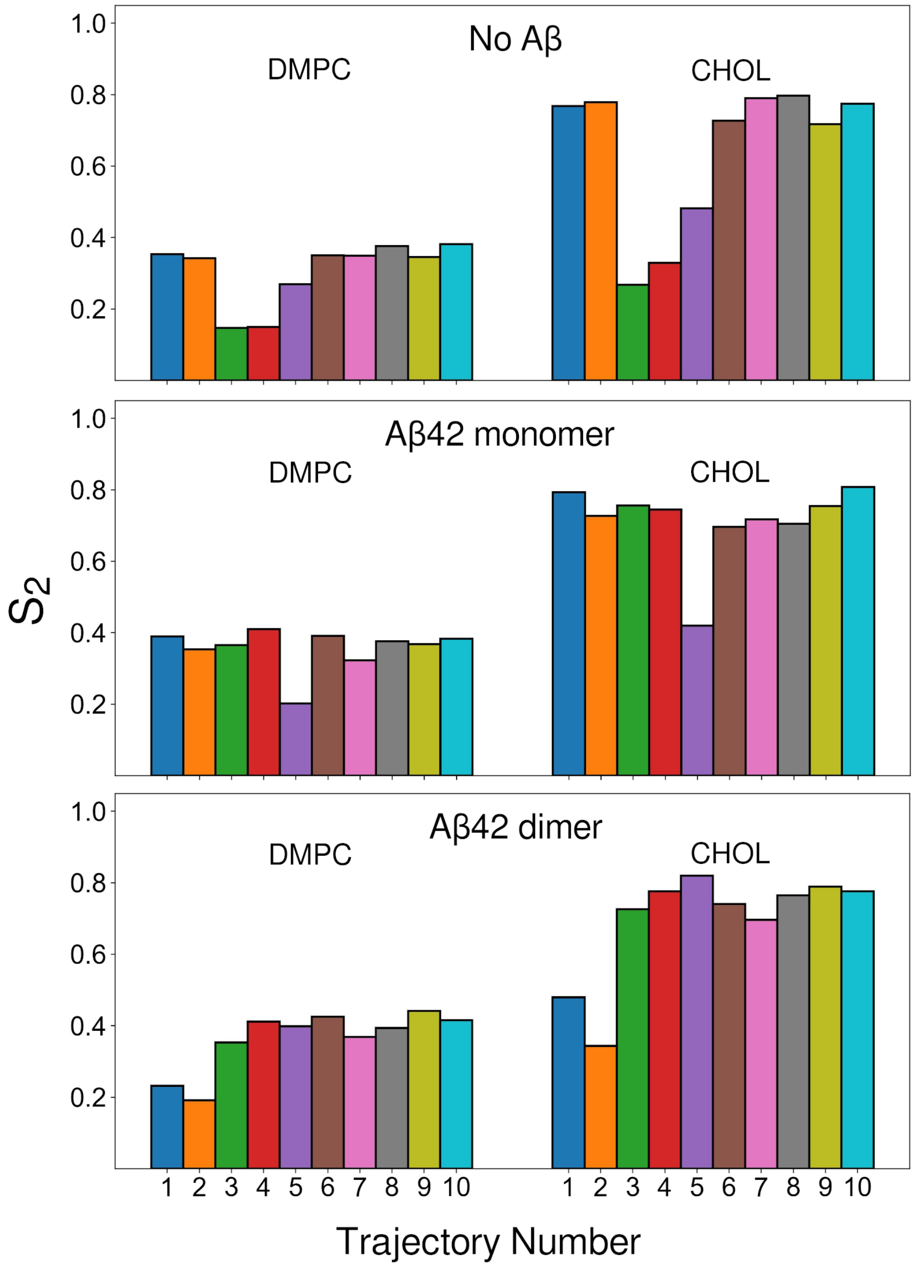

Fig. S13 The per-trajectory nematic order parameter for (A) lipids alone, (B) lipids with Aβ42 monomers, and (C) lipids with Aβ42 dimers, averaged over 400-500 ns of simulation time. For each of the three systems, different colors are used for ten replica trajectories.

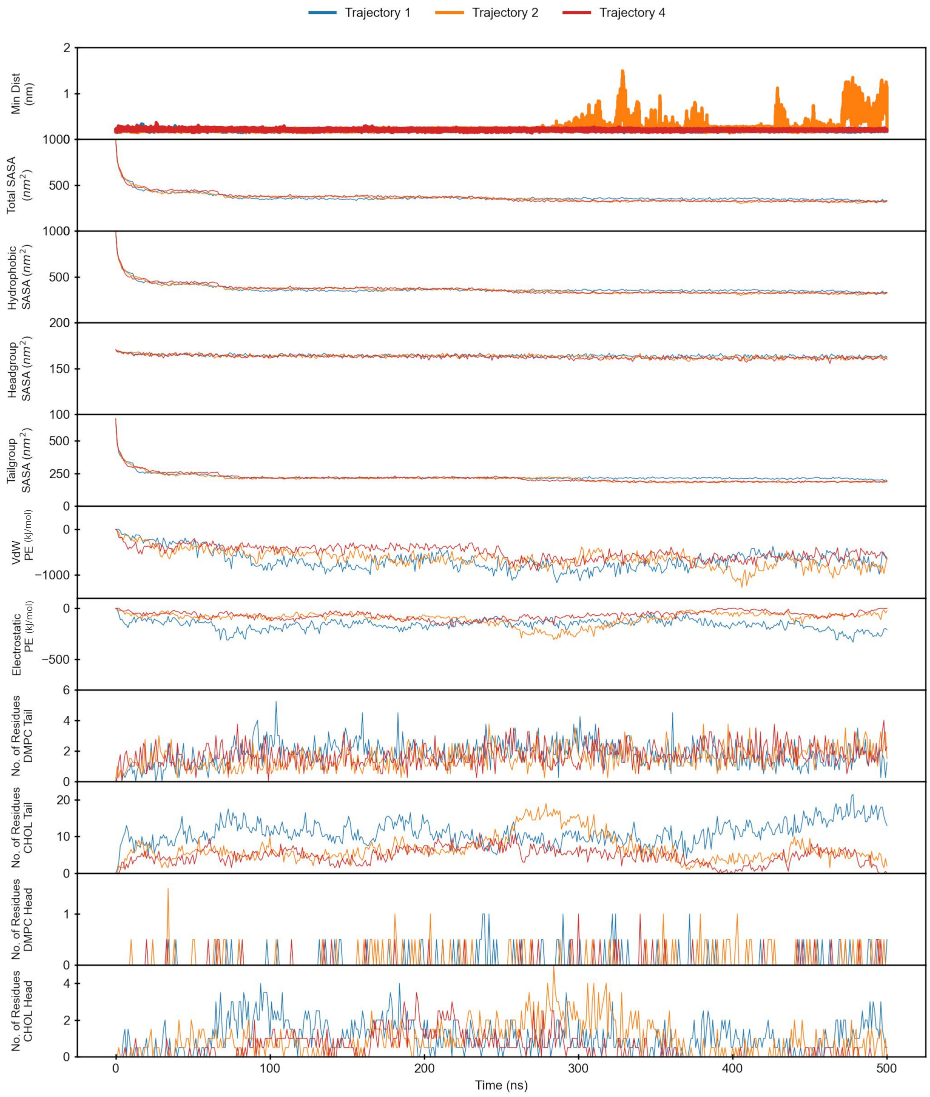

Fig. S14 Time evolution of eleven quantities of lipids-Aβ42 dimer system (v) for MD trajectories 1, 2, and 4. In the order from the top to the bottom, these quantities are: (1) the minimal distance between the two peptides in Aβ42 dimer, (2) the SASA and (3) the hydrophobic SASA of Aβ42 dimer, the SASA of (4) lipid headgroups and (5) lipid tailgroups, (6) van der Waals and (7) electrostatic energy of Aβ42 dimer-lipid system, and number of residues per peptide in contact with (8) DMPC tail groups, (9) CHOL tail groups, (10) DMPC head groups, and (11) CHOL head groups.

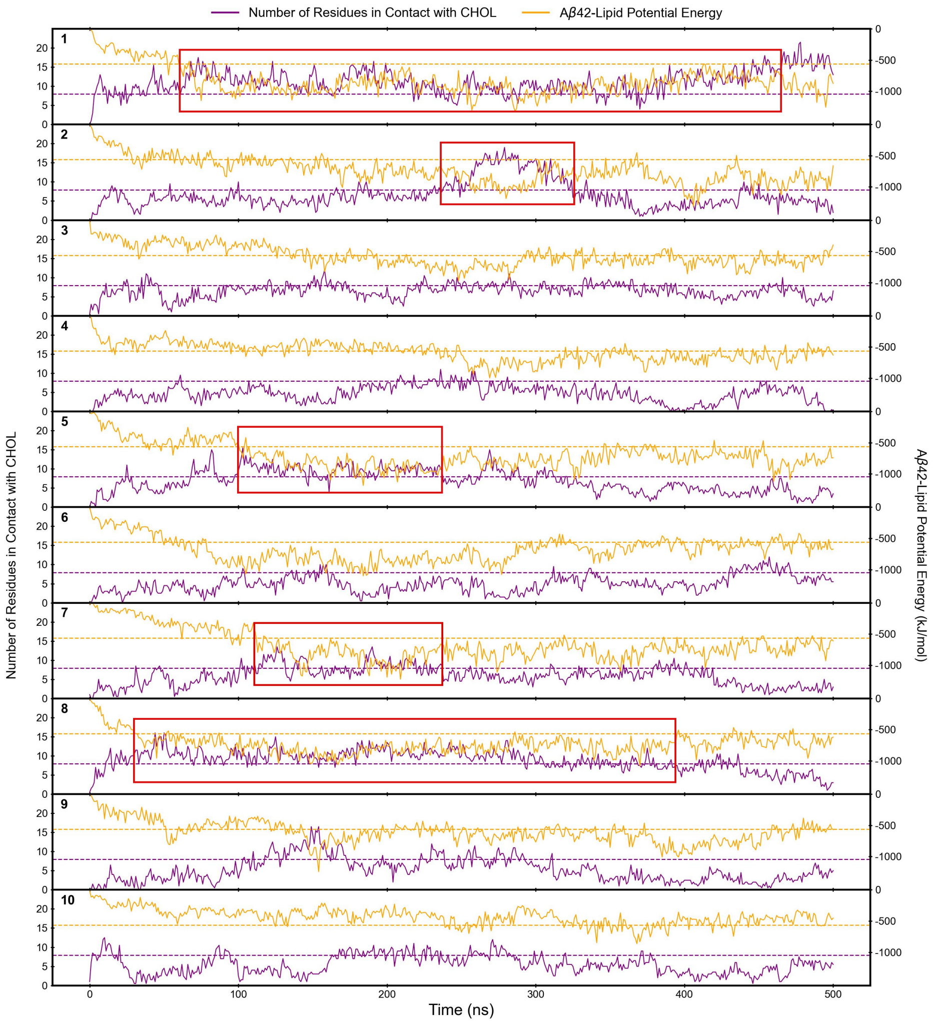

Fig. S15 Time evolution of the number of Aβ42 dimer residues in contact with CHOL tailgroups (purple) and the corresponding Aβ42 dimer-lipid interaction energy (orange), which is a sum of the van der Waals and electrostatic energy, for each of the ten MD trajectories of system (v). The purple and orange dashed lines correspond to the sum of the average and standard deviation of these two respective quantities calculated from the control trajectory 10, 7.94 and −563.08 kJ/mol, respectively, which are used as approximate threshold values for identifying the insertion events. The red rectangles correspond to all insertion events, longer than 50 ns.

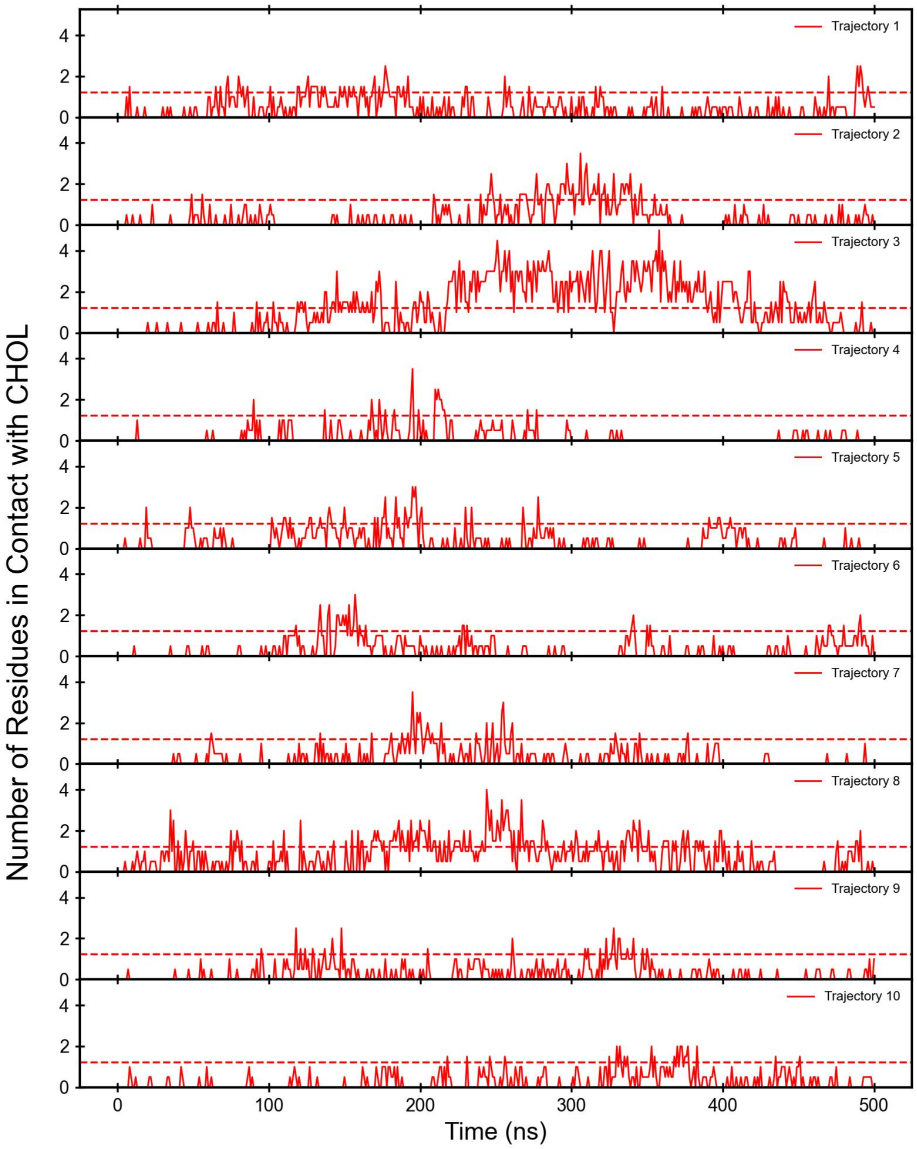

Fig. S16 The time evolution of the number of Aβ42 dimer residues in contact with CHOL head-groups (O-atom of CHOL) per peptide. The dashed line indicates the threshold value of 1.23, which is set to the sum of the average (0.5) and standard deviation (0.73) of this quantity over 50-500 ns of each trajectory, followed by an ensemble average over all 10 trajectories.

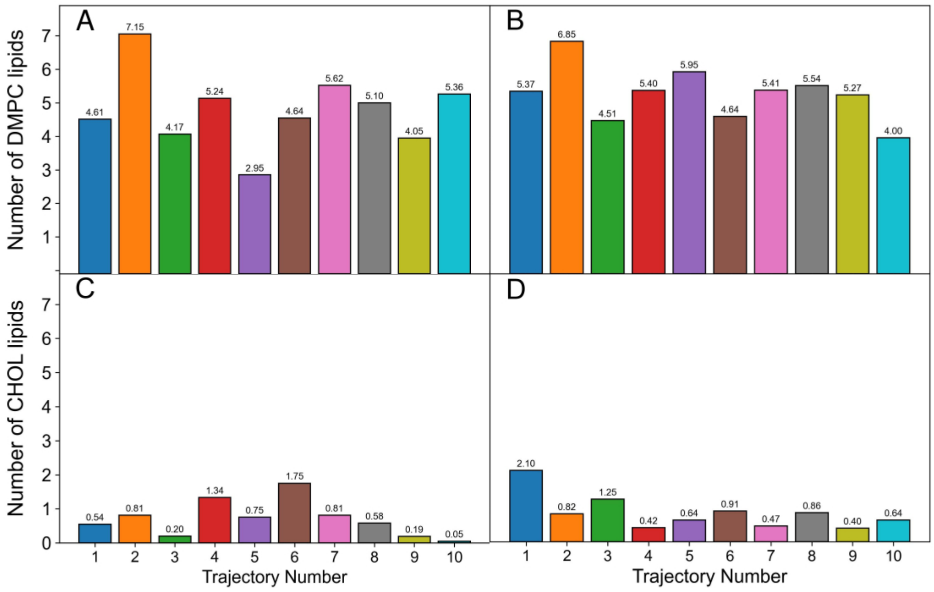

Fig. S17 The average number of (A-B) DMPC and (C-D) CHOL molecules in contact with (A,C) Aβ42 monomer and (B,D) one peptide of Aβ42 dimer for each of the ten replica trajectories. On average, Aβ42 monomer is in contact with 4.89 $\pm$ 0.33 DMPC and 0.70 $\pm$ 0.16 CHOL molecules. Each peptide in Aβ42 dimer is on average in contact with 5.29 $\pm$ 0.24 DMPC and 0.85 $\pm$ 0.15 CHOL molecules. Error bars correspond to SEM values, reflecting only trajectory-to-trajectory variability.
